# Selective decline of intact HIV reservoirs during the first decade of ART followed by stabilization in memory T cell subsets

**DOI:** 10.1097/QAD.0000000000004160

**Published:** 2025-02-20

**Authors:** Marieke M. Nühn, Kobus Bosman, Terry Huisman, Wouter H.A. Staring, Lavina Gharu, Dorien De Jong, Theun M. De Kort, Ninée V.E.J. Buchholtz, Kiki Tesselaar, Aridaman Pandit, Joop Arends, Sigrid A. Otto, Eduardo Lucio De Esesarte, Andy I.M. Hoepelman, Rob J. De Boer, Jori Symons, José A.M. Borghans, Annemarie M.J. Wensing, Monique Nijhuis

**Affiliations:** a Translational Virology, Department of Medical Microbiology, University Medical Center Utrecht; b Theoretical Biology, Utrecht University; c Center for Translational Immunology, University Medical Center Utrecht, Utrecht; d Department of Rehabilitation, Donders Institute for Brain, Cognition and Behaviour, Radboud University Medical Center, Nijmegen; e Faculty of Health, Medicine and Life Sciences, Maastricht UMC (MUMC), Maastricht; f Department of Internal Medicine and Infectious Diseases, University Medical Center Utrecht; g Translational Virology, Department of Global Public Health & Bioethics, University Medical Center Utrecht, Utrecht, The Netherlands.

**Keywords:** antiretroviral therapy, defective, HIV, intact, persistence, reservoir dynamics, T cell subsets

## Abstract

**Objectives:**

To investigate the short- and long-term dynamics of intact and defective proviral HIV DNA during ART.

**Design:**

We evaluated viral reservoir dynamics in a cohort of nine individuals with chronic HIV-1 subtype B who initiated first-line ART and were followed for 20 years while continuing ART.

**Methods:**

PBMCs were obtained before ART (*n* = 5), during the first year, and after 8.5 and 20 years of treatment. T cell subsets (naive, central-memory, transitional-memory and effector-memory) were sorted at 8.5 and 20 years. DNA was isolated and analyzed using the intact proviral DNA assay (IPDA). Deep-sequencing of the viral *env* region enabled analysis of viral evolution and cellular mechanisms underlying HIV persistence.

**Results:**

Initially, defective and intact proviral DNA in PBMCs declined with half-lives of 3.6 and 5.4 weeks, respectively. Over the following 8.5 years, the intact reservoir continued to decrease, with a half-life of 18.8 months in PBMCs, while defective proviral DNA levels stabilized. After 8.5 and 20 years of ART, the intact reservoir showed no further decline, with most intact proviral DNA residing in memory T cell subsets. Phylogenetic analysis revealed no signs of viral evolution over time, both within and between T cell subsets.

**Conclusions:**

PBMCs containing intact proviral DNA are selectively lost during the first decade of suppressive ART, followed by a decade of stabilization of this reservoir in the memory T cell subsets. In the absence of clear signs of viral evolution and massive clonal expansion, homeostatic proliferation might be an important driver of HIV persistence during long-term ART.

## Introduction

Shortly after HIV transmission, a genetically diverse viral population is established [1–4]. Upon initiation of suppressive antiretroviral therapy (ART), viral replication is halted [3,5–8]. However, even after years to decades on successful ART, a latent viral reservoir persists in CD4^+^ T cells [9–11], forming the major barrier for HIV cure.

Across multiple CD4^+^ T cell subsets, the most predominant HIV reservoir is found in resting CD4^+^ T cells [9,12–16]. Among these, naive cells exhibit the longest half-life and least differentiation, while more differentiated central memory (CM), transitional memory (TM), and effector memory (EM) cells have shorter half-lives and greater proliferative potential [14,17–20]. Consequently, each T cell subset likely contributes differently to persistence of the viral reservoir [14,21]. Generally, HIV frequency is highest in the memory CD4^+^ T cell subsets [12–15,22]. However, it remains elusive whether the differentiation of naive and/or memory cells towards more differentiated memory subsets causes shifts in reservoir sizes and constitution across subsets of T cells, especially after decades of ART.

HIV proviral DNA is primarily composed of defective proviruses [23,24], with only a small fraction of intact proviruses capable of producing replication competent virus [25], which likely triggers viral rebound after cessation of ART [26]. However, defective proviral DNA can still produce viral transcripts, proteins and even noninfectious viral particles, contributing to chronic immune activation [27–31]. Therefore, measuring both intact and defective proviral DNA is imperative to advance our understanding of HIV persistence.

The intact proviral DNA assay (IPDA) is a multiplex droplet digital PCR (ddPCR) using two specific amplicons to reliably distinguish between intact and defective proviruses [23,32]. Several studies have employed this method to investigate proviral DNA dynamics during either the initial or late phases of ART. It has shown a sharp decline in the sizes of intact and defective proviral DNA during the first months of ART [33,34]. Other studies reported a gradual, slower decline of the intact reservoir over the following years [10,35–39], while defective proviral DNA stabilizes after the initial rapid decay [35,36,39]. After the biphasic decline of the intact reservoir during the first decade of ART, the decline eventually levels off [36,40], emphasizing the need for lifelong adherence to ART. However, the distribution of intact and defective proviral DNA in an individual over time remains unclear, as none of these studies have examined the dynamics of proviral DNA during both the early (weeks, months) and late phases (decades) of ART. Furthermore, the influences of cellular mechanisms such as immunologic memory, homeostatic proliferation and clonal expansion as drivers of HIV persistence over time and across different subsets are not well understood. Unraveling these reservoir dynamics would enhance our understanding of long-term HIV persistence and inform the design of novel HIV cure strategies. To address this, we used the IPDA and viral sequencing to characterize the early and late dynamics of proviral DNA in a unique cohort of individuals who initiated their first-line ART over 20 years ago and from whom we have been able to obtain frequent (large) blood-draws.

## Methods

### Participant inclusion criteria

Nine individuals with chronic HIV-1 who started ART in 1997 or 1998 as part of a randomized clinical trial comparing two protease inhibitor (PI)-based regimens were included [41]. Selection criteria included: treatment-naive at ART initiation, availability of longitudinal peripheral blood mononuclear cell (PBMC) samples over 18–20 years of ART, and no signs of virological rebound during the study.

### Ethical approval

The study was approved by the institutional ethical review board (98093, METC-04017, NL55494.041.16/METC16-226). All participating individuals gave written informed consent.

### Blood sample processing

Blood was obtained at multiple timepoints during the first year after start of ART. Additionally, for most individuals one or two large (500 ml) blood draws were obtained 7–20 years after ART initiation. PBMC were isolated by Ficoll gradient centrifugation. From the large blood draws, CD4^+^ T cells were isolated using negative selection (MACS CD4^+^ T cell isolation kit; Miltenyi Biotec). CD4^+^ T cells were subsequently sorted using FACS (BD Biosciences) into naive (CD27^+^ CCR7^+^ CD45RO^−^ Fas^−^), central memory (CD27^+^ CCR7^+^ CD45RO^+^ Fas^+^), transitional memory (CD27^+^ CCR7^−^ CD45RO^+^ Fas^+^), and effector memory (CD27^−^ CCR7^−^ CD45RO^+^ Fas^+^) cells.

### Nucleic acid isolation

DNA was extracted from 1–5 million PBMCs or CD4^+^ T cells (DNeasy Blood and Tissue kit; Qiagen). Baseline plasma RNA was performed (RNeasy Mini Kit; Qiagen).

### Plasma viral load

Plasma HIV-1 quantifications were performed during clinical monitoring. In the year 2000, technical advancements lowered the limit of detection of this assay from 400 to 50 HIV-1 RNA copies/ml.

### Droplet digital PCRs

Droplet digital PCR (ddPCR) was conducted for both the HIV-1 long terminal repeat (LTR) assay and the IPDA using the QX200 Droplet Digital PCR system (Bio-Rad). Droplets were created using the Droplet Generator and ddPCR Droplet Reader Oil (Bio-Rad), combined with ddPCR Supermix for probes (no dUTP) (Bio-Rad), primers, probes, and a specific restriction enzyme (details below). All samples were analyzed in duplicate. PCR was performed in a T100 thermal cycler (Bio-Rad) under the conditions specified in Table 1, Supplemental Digital Content, and droplets were assessed using the QX200 droplet reader. Only samples with >10 000 readable droplets were included in the analysis. Results were analyzed using Quantasoft version 1.7.4, with positive and negative droplets identified through manual thresholding [42].

### HIV-1 LTR quantification

A maximum of 500 ng DNA from each sample was digested with EcoRI (10U/μl, buffer H; Roche) at 37°C for 1 h. The number of cells as input into the assay was determined from 10% of the digestion mixture using the cellular RPP30 gene [43]. The remaining sample was mixed with HIV LTR primers and probes, as detailed in Table 2, Supplemental Digital Content [44,45]. DNA from PBMCs of HIV-negative donors served as a template control, water as a no-template control, and U1 cells as an HIV-positive control (NIH AIDS Reagent Program).

### IPDA

The multiplex ddPCR IPDA was performed as previously described [23,32]. Proviral DNA was quantified by targeting the Ψ (*psi*) and *env* region. The DNA Shearing Index (DSI) and input cell number were calculated using the cellular RPP30 gene. A maximum of 500 ng of DNA was used for the *psi/env* assay, and 20ng DNA for the RPP30 assay. The restriction enzyme XhoI (20U/μl, NEB) was added directly to the DNA, primers and probe mix. Primers and probe concentrations and conditions were described previously [32]. PBMC DNA of HIV-negative donors served as a template control, water as a no-template control, and DNA from J-Lat full-length clone 15.4 as an HIV-positive control (NIH AIDS Reagent Program). Samples were included only if: DSI <0.50, and >100 000 cells were analyzed and/or more than the limit of detection (LoD) of 6 intact and 7 defective HIV proviral DNA copies was detected [32]. If fewer copies were detected, these were left censored and visualized using a different symbol to denote quantification below the LoD [32]. The total of single positive *env* and *psi* copies was reported as the number of defective copies.

### HIV envelope amplification and sequencing

Participant-derived RNA and DNA was amplified in Duplo in a nested PCR approach to obtain the HIV-1 *env* region which contains all five variable loops of glycoprotein gp120 (1106 bp product). Primers and cycling conditions are as detailed in Tables 3 and 4, Supplemental Digital Content. For RNA, the outer PCR was performed using the Titan One Tube RT-PCR kit (Roche), while for DNA, the outer amplifications used the Platinum Taq High Fidelity DNA Polymerase kit (Thermo Fisher Scientific) both with maximum 1 μg input. Inner PCRs were performed using the Expand High Fidelity kit (Roche) and 2.5 mg outer PCR product. Subsequently, PCR products were cleaned (QiaQuick PCR purification kit; Qiagen) and sequenced (MiSeq v2 reagent kit (500 cycles)) to yield paired end reads of 250 bases each. Reads were aligned to the consensus HIV-1B sequence and those that overlapped the entire V3 region were isolated and trimmed. Unique V3 sequences supported by less than 2% of the total V3 read count in a sample were excluded, to minimize the impact of potential PCR or sequencing errors, or carry-over contamination from prior MiSeq runs. Subsequently, replicate samples were pooled.

### Tropism prediction

After filtering the V3 sequences, co-receptor tropism was predicted using geno2pheno [46–49]. The weighted occurrence of each unique V3 amino acid sequence was considered when evaluating the false positive rate (FPR).

### Phylogenetics

Five random starting phylogenetic trees of V3 sequences were aligned by hand [50]. Maximum likelihood searches were performed under the General Time Reversible (GTR) model in PhyML 3.1, applying Nearest Neighbor Interchange (NNI) and Subtree Pruning and Regrafting (SPR) [51]. The tree with the highest maximum likelihood was selected for further analysis and branch support was evaluated through 100 bootstrap iterations.

### Calculations, statistical assays, and graphical visualization

For statistical analyses and visualization of ddPCR data, samples were grouped based on timepoint. The median half-life of the proviral DNA reservoir was calculated for different time frames by determining each individuals half-life (ln(2)/decay constant) and then taking the median of these values. Statistical analyses were conducted in R 4.1.2. Paired analyses were conducted using the Wilcoxon signed-rank test. For *P*-values < 0.1, additional Mann–Whitney *U* tests were performed to include unpaired samples. *P*-values < 0.05 were considered significant. Group comparisons were performed using Kruskal–Wallis tests, followed by Dunn's posthoc tests with Bonferroni correction, with statistical significance defined as *P* < 0.05. Statistical methods were also detailed in the figure legends. Graphs were composed using R 4.1.2.

## Results

### Cohort characteristics

Nine individuals with chronic subtype B HIV-1 started first-line ART in 1997 or 1998. At baseline, their median CD4^+^ T cell count was 160 cells/mm^3^, and the median viral load was 7 × 10^4^ HIV RNA copies/mL (Table 1). Individuals were considered virally suppressed with viral loads below limit of detection (at start of the study <400 copies/ml, after the year 2000 below 50 copies/ml). During the study period, the median CD4^+^ T cell count increased to >900 cells/mm^3^. HIV suppression was achieved in all individuals within 16 weeks of treatment (Fig. 1 a). Three individuals remained suppressed throughout the study (participants 1, 5, 7). One participant experienced several viral blips within the first 100 weeks (participant 9), while four others demonstrated 1–2 viral blips during 20 years of treatment (participants 2, 3, 6, 8). Two individuals were excluded after demonstrating high viral loads: one discontinued ART after 5.7 years (participant 4), and the other experienced a viral rebound of >10^5^ RNA copies/ml after 14.6 years of ART (participant 9), (Fig. 1 a). The ART regimen details, and major clinical events are included in Table 5, Supplemental Digital Content. No additional treatment with immunomodulators was reported.

**Table 1:** Overview of the characteristics of the cohort.

ID	Gender	ART initiation				7–10 years after start therapy					18–20 years after start therapy				
		Age	CD4^+^ cell count (cells/mm^3^)	CD4/CD8 ratio	Viral load (copies/ml)	Age	Time on ART (years)	CD4^+^ cell count (cells/mm^3^)	Viral load (copies/ml)	Large blood draw?	Age	Time on ART (years)	CD4^+^ cell count (cells/mm^3^)	Viral load (copies/mL)	Large blood draw?
1	M	38	30	0.17	5.E+05	47	8.6	487	<50	No	59	18.9	656	<50	Yes
2	M	51	160	0.09	2.E+05	59	8.5	836	<50	Yes	70	20.3	948	<50	Yes
3	M	41	320	0.20	7.E+04	50	8.5	883	<50	Yes	61	20.3	1290	<50	Yes
4	M	29	30	0.24	2.E+06	Exclusion					Exclusion				
5	M	37	440	0.78	9.E+03	44	7.6	924	<50	Yes	56	18.9	847	<50	Yes
6	M	40	590	0.73	7.E+04	49	8.8	1361	<50	Yes	60	20.3	1085	<50	Yes
7	M	31	380	0.51	3.E+04	40	8.8	1267	<50	Yes	51	20.3	1052	<50	Yes
8	M	36	50	0.16	3.E+04	44	7.9	664	<50	No	56	19.9	700	<50	No
9	M	45	30	0.61	2.E+05	52	7.1	1080	<50	Yes	Exclusion				
	**Median [IQR]/summary:**	**38 [36–40]**	**160 [30–380]**	**0.24 [0.17–0.61]**	**7.E+04 [3.E+04–2.E+05]**	**48 [44–51]**	**8.5 [7.8–8.7]**	**904 [793–1127]**	**<50**	**6/8**	**59 [56–61]**	**20.3 [19.4–20.3]**	**948 [774–1069]**	**<50**	**6/7**

Median values including the interquartile range (IQR) or a summary of the values are given in the last row.

**Fig. 1:**
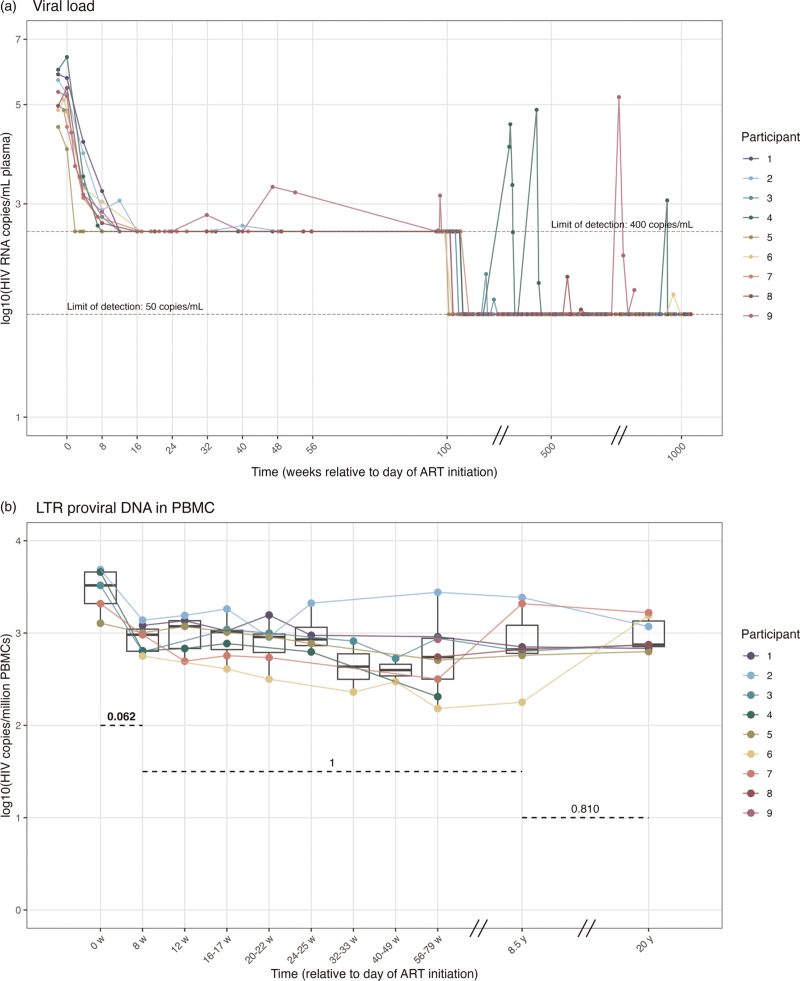
Dynamics of HIV viral load (A) and proviral DNA (B-D) during two decades of ART.

A) Overview of viral load data during study period. Viral load is considered suppressed when below the limit of detection of 400 copies/mL and later of 50 copies/mL. These limits of detection are indicated with dotted lines. Because of large viral rebounds, individual 9 was excluded after 8.5 years and individual 9 after 1 year of ART. B) Size of the LTR proviral DNA as measured via the ddPCR assay. Size of the C) defective and D) intact proviral DNA as measured via the IPDA. The sum of the psi and *env* proviral copies are considered as the total number of defective proviral DNA copies, dynamics of psi and *env* separately are shown in Supplementary Figure 1. Data points below the lower bound of detection (open squares) or quantified with less than 95% reliability (triangles) are indicated. Horizontal x-axis is not in scale; “w” represents time points weeks, “y” represents years after ART. Statistical comparisons of the Wilcoxon singed-rank tests are shown. With P-values <0.1, these comparisons were additionally tested with a Mann-Whitney U test (unpaired). P-values of the Wilcoxon singed-rank <0.1 and the Mann-Whitney U test <0.05 are indicated in bold.

**Fig. 1 (Continued):**
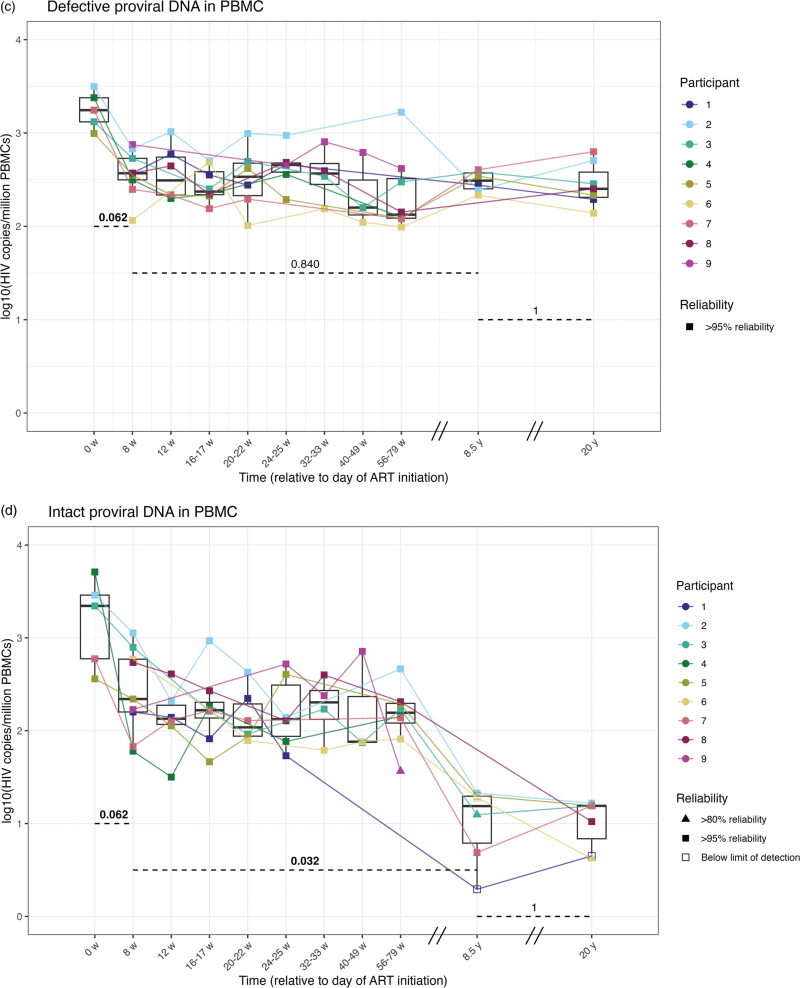
Dynamics of HIV viral load (A) and proviral DNA (B-D) during two decades of ART.

A) Overview of viral load data during study period. Viral load is considered suppressed when below the limit of detection of 400 copies/mL and later of 50 copies/mL. These limits of detection are indicated with dotted lines. Because of large viral rebounds, individual 9 was excluded after 8.5 years and individual 9 after 1 year of ART. B) Size of the LTR proviral DNA as measured via the ddPCR assay. Size of the C) defective and D) intact proviral DNA as measured via the IPDA. The sum of the psi and *env* proviral copies are considered as the total number of defective proviral DNA copies, dynamics of psi and *env* separately are shown in Supplementary Figure 1. Data points below the lower bound of detection (open squares) or quantified with less than 95% reliability (triangles) are indicated. Horizontal x-axis is not in scale; “w” represents time points weeks, “y” represents years after ART. Statistical comparisons of the Wilcoxon singed-rank tests are shown. With P-values <0.1, these comparisons were additionally tested with a Mann-Whitney U test (unpaired). P-values of the Wilcoxon singed-rank <0.1 and the Mann-Whitney U test <0.05 are indicated in bold.

### Continuous decline of the intact viral reservoir over the first decade of ART

The rapid initial decline in plasma viral RNA is typically associated with a decline of proviral DNA [11]. We quantified HIV LTR copies, a common marker for total proviral DNA load [45]. Initiation of ART resulted in a sharp decline in HIV LTR copies within 8 weeks in PBMCs of all five individuals with sufficient cells available for IPDA baseline analyses (Fig. 1 b). After this initial rapid decline, HIV LTR copies remained relatively stable over the following years and decades (Fig. 1 b, Table 2). Despite undetectable plasma viral RNA levels, the HIV LTR copy number persisted, with median levels remaining consistent, ranging from a minimum median of 414 and a maximum of 1183 copies per million cells (Fig. 1 a, b, Table 2).

**Table 2:** Overview of the total proviral DNA copies and their half-lives at different moments after start of ART.

	LTR (total)	Defective proviral DNA	Intact proviral DNA
**Copies proviral DNA per million cells: median [IQR]**			
Baseline	3287 [2084–4585]	1757 [1318–2389]	2211 [596–2888]
8 weeks	960 [636–1108]	371 [315–535]	220 [159–590]
12 weeks	1183 [681–1362]	331 [217–556]	135 [117–191]
16–17 weeks	1026 [670–1072]	236 [219[389]	166 [142–205]
20–22 weeks	908 [632–979]	347 [216–477]	110 [87–199]
24–25 weeks	859 [735–1237]	455 [383–478]	134 [89–338]
32–33 weeks	524 [377–671]	369 [296–497]	205 [144–279]
40–49 weeks	414 [356–473]	159 [135–389]	76 [75–396]
1 year	549 [317–878]	134 [122–328]	157 [124–197]
8.5 years	665 [605–1396]	311 253 [374]	16 [7–20]
20 years	750 [715–1365]	252 [205–397]	16 [8–16]
**Decline over time: half-life (*P*-value)**			
0–8 weeks	4.4 weeks **(*P* = 0.062/0.005)**	3.6 weeks **(*P* = 0.062/0.001)**	5.4 weeks **(*P* = 0.062/0.029)**
8 weeks–8.5 years	+14.6 months (*P* = 1)	138.0 months (*P* = 0.840)	18.8 months **(*P* = 0.031/<0.001)**
8.5 years–20 years	+44.2 months (*P* = 0.810)	209.3 months (*P* = 1)	9.7 months (*P* = 1)

The half-life of proviral DNA was determined by calculating the half-life (ln(2)/decay constant) for each individual and subsequently reporting the median of these half-lives. Statistical comparisons of the Wilcoxon singed-rank tests are shown. When below <0.1, these comparisons were additionally tested with a Mann–Whitney *U* test (unpaired) shown as second *P*-value. *P*-values of the Wilcoxon singed-rank <0.1 and the Mann–Whitney *U* test <0.05 considered significantly different are indicated in bold.

The IPDA enabled precise quantification of defective proviral DNA and the intact HIV reservoir. Proviruses with 5’ deletions (env) and 3’ deletions (psi) behaved similarly and were therefore considered as total defective proviral DNA, Figure 1, Supplemental Digital Content, Fig. 1 c. ART initiation resulted in a sharp decline in defective HIV copies in PBMCs within 8 weeks in PBMCs of all 5 individuals with sufficient cells available for IPDA baseline analyses, with a half-life of 3.6 weeks (Table 2), and remained stable afterward at a median level of 311 copies per million cells at 8.5 years and 252 copies per million cells at 20 years. The dynamics of defective proviral DNA mirrored those of HIV LTR DNA (Fig. 1 c, Table 2), as expected, since total proviral DNA is predominantly composed of defective viruses [23,24].

During the first 8 weeks following ART initiation, the decay dynamics of the intact reservoir closely mirrored those of total and defective proviral DNA (Fig. 1 d, Table 2). The half-life of the intact proviral reservoir was 5.4 weeks during the first 8 weeks of ART. Consequently, the *relative* fraction of the intact reservoir remained stable in PBMCs during these first 8 weeks of treatment (Figure 2, Supplemental Digital Content). Unlike the defective proviral DNA, the initial sharp decline was followed by a slower decline of the intact proviral reservoir in PBMCs, averaging a half-life of 18.8 months from 8 weeks to 8.5 years after ART initiation, resulting in a decrease in the relative fraction of intact proviral DNA (Figure 2, Supplemental Digital Content). During the second decade, the decline in the intact reservoir further leveled off and became stable in PBMCs at median levels of 16 copies per million cells at both 8.5 years and 20 years after treatment initiation (Table 2). These data indicate that while the defective proviral DNA stabilizes after the first 8 weeks of ART in PBMCs, the intact reservoir continues to decline throughout the first decade but demonstrates long-term persistence into the second decade.

### The intact viral reservoir is enriched within memory T cell subsets after decades of ART

Persistence of the intact viral reservoir in PBMCs prompted an investigation into which specific cellular CD4^+^ T cell subsets predominantly harbor HIV and whether T cell proliferation and differentiation affects this distribution over time. We sorted naive, CM, TM, and EM cells from the large blood draws at 8.5- and 20-years posttreatment initiation (Table 1) and measured proviral DNA. Intact and defective proviral DNA could be detected in all T cell subsets at 8.5- and 20-years posttreatment (Fig. 2 a, b). When comparing reservoir sizes between T cell subsets at 20 years posttreatment, both intact and defective proviral DNA levels were significantly higher in the memory CM and EM cells compared to the naive cells. Enrichment in the memory TM cells was also visually apparent, although this was not statistically significant (Fig. 2 a, b). Over time, no clear shifts were observed in the level of defective proviral DNA or the intact viral reservoir among the different T cell subsets, except for a decrease in the intact reservoir within the TM subset. The proportion of the intact viral reservoir represented 10% of the total proviruses and was relatively higher in the naive cells compared to the PBMCs and memory subsets. This is among others demonstrated by the significant enrichment in the EM cells compared to the naive cells at 8.5 years after treatment initiation **(**Fig. 2 c).

**Fig. 2:**
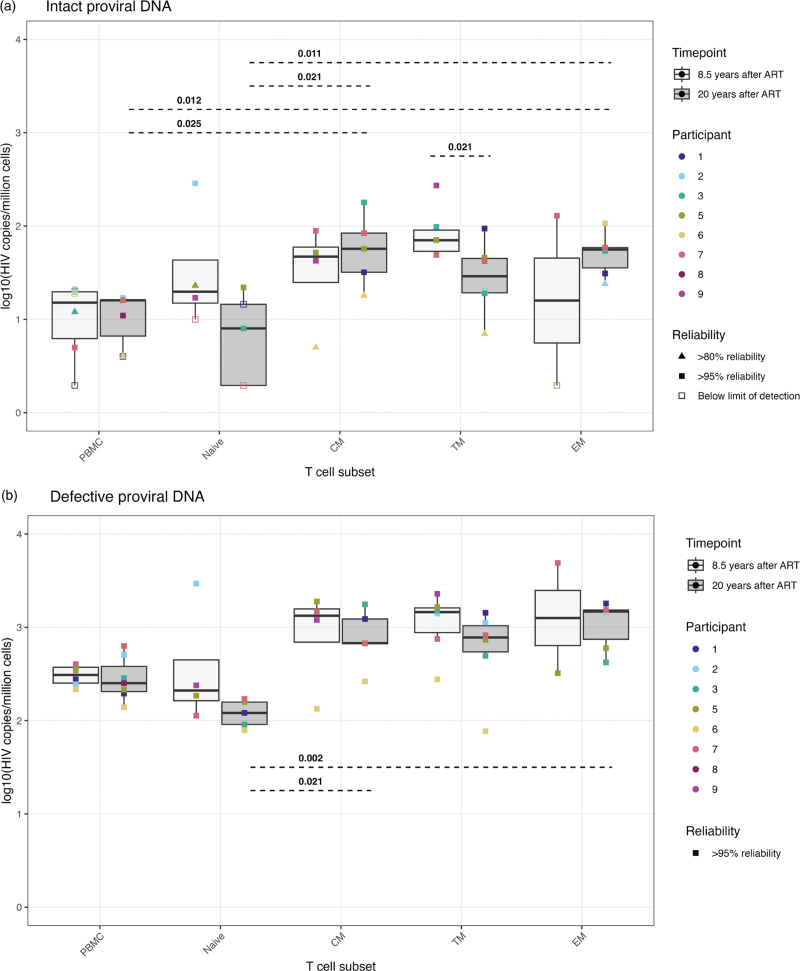
The absolute and relative size of the intact and defective proviral DNA in PBMC and in CD4^+^ T cell subsets after 8.5 and 20 years of ART.

Size of the A) intact and B) defective proviral DNA was measured using the IPDA. The sum of the psi and *env* proviral copies are considered as the total number of defective proviral DNA copies. C) The relative size of the intact proviral reservoir was defined as the size of the intact reservoir as a percentage of the total proviral DNA. Data points below the lower bound of detection (open squares) or quantified with less than 95% reliability (triangles) are indicated. Statistical comparisons between different T cell subsets were performed using the Kruskal-Wallis test, with a Dunn's post-hoc test. Only P-values <0.05 are given in the plots. Comparisons between timepoints were tested with a Wilcoxon signed-rank tests. With P-values <0.1, these comparisons were additionally tested with a Mann-Whitney U test (unpaired). Only P-values of the Wilcoxon singed-rank <0.1 and the Mann-Whitney U test <0.05 are shown.

**Fig. 2 (Continued):**
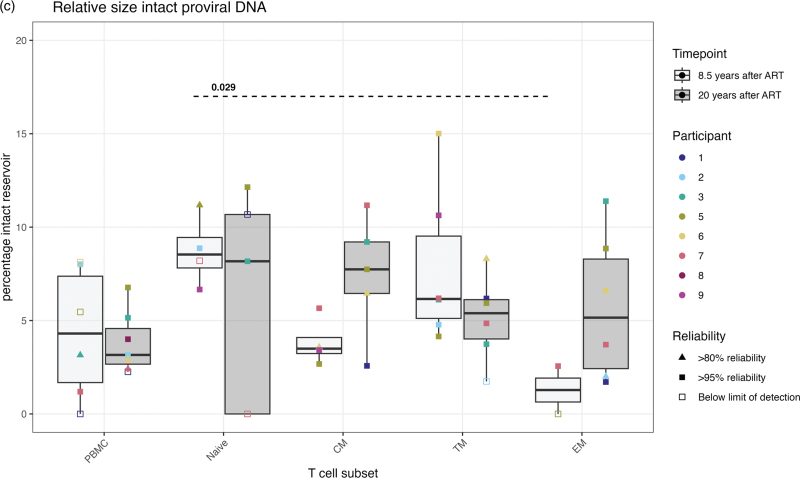
The absolute and relative size of the intact and defective proviral DNA in PBMC and in CD4^+^ T cell subsets after 8.5 and 20 years of ART.

Size of the A) intact and B) defective proviral DNA was measured using the IPDA. The sum of the psi and *env* proviral copies are considered as the total number of defective proviral DNA copies. C) The relative size of the intact proviral reservoir was defined as the size of the intact reservoir as a percentage of the total proviral DNA. Data points below the lower bound of detection (open squares) or quantified with less than 95% reliability (triangles) are indicated. Statistical comparisons between different T cell subsets were performed using the Kruskal-Wallis test, with a Dunn's post-hoc test. Only P-values <0.05 are given in the plots. Comparisons between timepoints were tested with a Wilcoxon signed-rank tests. With P-values <0.1, these comparisons were additionally tested with a Mann-Whitney U test (unpaired). Only P-values of the Wilcoxon singed-rank <0.1 and the Mann-Whitney U test <0.05 are shown.

### No clear signs of viral evolution or clonal expansion as major drivers of HIV persistence during two decades of ART

**Fig. 3:**
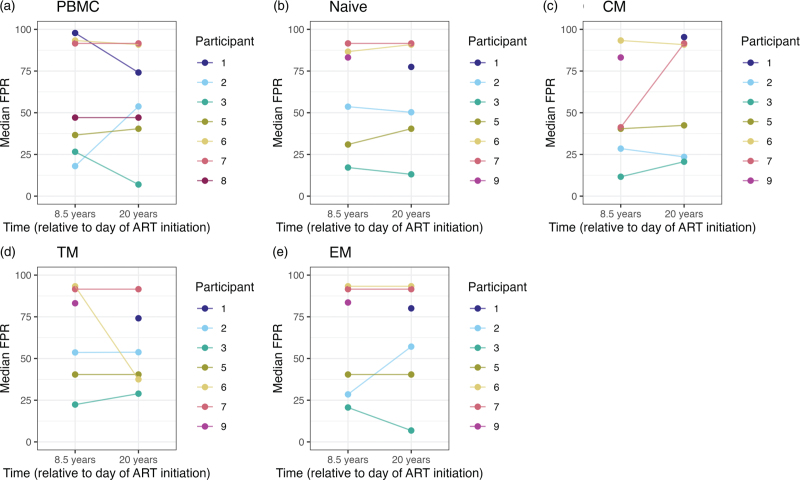
Median FPR for (A) PBMC and for (B) naive, (C) CM, (D) TM and (E) EM CD4^+^ T cell subsets.

To understand the mechanisms underlying HIV persistence during long-term ART, we sequenced the highly variable part of the viral envelope to evaluate the role of ongoing viral replication and evolution. Additionally, we used the HIV sequences to gain insights into the role of clonal expansion and T cell differentiation in long-term viral persistence. HIV envelope sequences were obtained at baseline (plasma viral RNA and PBMCs), at several timepoints during the first years of ART (PBMCs), and after 8.5 and 20 years (PBMCs and T cell subsets). To explore the role of T cell differentiation in HIV persistence, we investigated viral coreceptor usage. Naive T cells express in addition to the CCR5 coreceptor also CXCR4, while memory T cells predominantly express CCR5 [52–55], leading to infection by dual/X4-tropic and R5-tropic viruses, respectively [52,56]. We used the geno2pheno prediction algorithm [46–48] to assess coreceptor preference of the *env* sequences from the different T cell subsets at 8.5 and 20 years after ART initiation (Figure 3, Supplemental Digital Content **)**. Remarkably, median FPR values showed a consistent pattern across all individuals and did not differ significantly between naive and memory T cell subsets (Fig. 3). Consequently, FPR values cannot be used to determine whether naive T cells harboring dual/X4 tropic HIV have differentiated into memory T cells.

Each individual is indicated by a different color. The weighted occurrence of each unique V3 amino acid sequence was considered when evaluating the FPR. The median FPR value of all *env* sequences within a sample were plotted. Paired comparisons between timepoints were tested with a Wilcoxon signed-rank test, none of them were significant.

Subsequently, we investigated the relatedness of the viral *env* sequences in a phylogenetic tree. We did not observe clear signs of viral evolution as sequences from the late timepoints (8.5 and 20 years) were scattered among those from baseline and early treatment (Figure 4, Supplemental Digital Content). Additionally, we did not observe large sequence clusters in PBMCs or specific T cell subsets over time, suggesting massive clonal expansion of activated T cells is unlikely to be the major driver of viral persistence in these individuals.

## Discussion

We studied HIV reservoir dynamics over two decades of ART in nine individuals. While prior studies either lacked sampling before ART [10,35–40] or investigated HIV dynamics during the first year of ART [33,34], we sampled before and during early and long-term ART and in subsets of CD4^+^ T cells. Initially, during the first few months of ART, both intact and defective proviral DNA declined sharply, with the defective proviral DNA stabilizing and the intact viral reservoir continuing to decline slowly over the first decade in PBMCs. During the second decade, both the defective proviral DNA and the intact reservoir remained stable and were predominantly present in memory CD4^+^ T cells. We found no evidence for ongoing viral evolution and/or replication, nor did we observe a clear role for clonal expansion driving T cell proliferation and persistence of the reservoir during long-term ART.

Over the past years, several studies have used the IPDA to examine and compare the dynamics of the intact reservoir following ART initiation. These dynamics are influenced by multiple clinical factors, including the timing of ART initiation, the ART regimen used, and the timing of sampling during ART. Within this study, ART was initiated 20 years ago in ART-naive individuals, at a time when PI-based regimens were the standard treatment, and diagnosis and treatment initiation occurred later during chronic infection. We observed an initial rapid decay with a half-life of 5.4 weeks during the first 8 weeks of ART in PBMCs. This was followed by a slower decline with a half-life of 18.8 months in PBMCs during 8.5 years of ART. Recent studies by White *et al.* and Barbehenn *et al.* have examined the early dynamics of the intact reservoir in individuals on more modern integrase (INSI-)based ART regiment [33,34]. White *et al.* reported an initial phase with a half-life of 12.9 days during the first 3 months of ART, followed by a slower phase with a half-life of 19 months for the remainder of the year. In their study, 71% of the individuals were also ART naive, while 29% had interrupted ART of >6 months prior to inclusion [33]. Barbehenn *et al.*, on the other hand, included PWH during acute infection (within 100 days of infection), and observed a decay with a half-life of 2.83 weeks (0.7 months) during the first 5 weeks of ART. This was followed by a slower decline with a half-life of 15.4 weeks (3.9 months) during the subsequent half-year of ART [34]. These faster early half-lives reported by White *et al.* and Barbehenn *et al.* (12.9 days and 2.83 weeks, respectively), may be explained by the earlier ART initiation [34] and the use of INSTI-based regimens [57], which are known to promote a more rapid decline of the HIV reservoir as compared to PI-based regiments. Differences in the measured timeframes may also contribute to the observed variations, as our study examined dynamics over the first 8 weeks, while these studies focused on earlier or shorter intervals.

For the next phase of intact reservoir decline, White *et al.* and Barbehenn *et al.* measured half-lives beginning at 5 weeks or 3 months and extending through the remainder of the (half) year [33,34]. In contrast, our second phase of decline, with a half-life of 18.8 months, spanned from 8 weeks to 8.5 years of ART. Other studies, such as those by Peluso *et al.* and McMyn *et al.* reported much longer half-lives (44–46 months) during the median period from 617 days to 7 years after ART initiation [35,36]. Similarly, Gandhi *et al.* observed a median half-life of 7.1 years in treatment-naive individuals at start of the study, during a timeframe of 7.1–12 years post treatment initiation [39]. All three studies included participants based on PI- and integrase-based regimens in a chronic infection. The differences in reported half-lives are likely influenced on the specific timeframes studied, apart from the clinical cohort characteristics. Additionally, it is important to note that the studies referred to here measured reservoir dynamics in peripheral CD4^+^ T cells, whereas our study used PBMCs. This distinction may have implications for the observed half-lives, as distinct cell-type dynamics could affect reservoir dynamics [58]. These differences in cellular characteristics complicate direct comparisons across our study and these studies. Despite these differences, our findings combined with findings of prior studies support the hypothesis of a continuous decline of the reservoir over the first decade of ART, with the rate of decline progressively slowing over time. Multiple studies suggest that this decline of infected cells is attributed to viral cytopathic effects (CPE) or host immune surveillance [33,35,39].

Along these lines, during the initial phase of 8 weeks on ART, cells harboring defective proviral DNA decay with a half-life of 3.6 weeks in PBMCs, comparable to the decline of the intact reservoir, as reflected by the stable relative fraction of the intact reservoir over this period. Interestingly, Barbehenn *et al.*, observed an even faster decline of the defective reservoir in acutely treated individuals on INSTI-based regimens [34]. In contrast, Reddy *et al.* reported a faster initial decline of the intact reservoir (51% per month) compared to the defective proviral DNA (35% per month) in hyperacute treated individuals (1–3 days postinfection) also on INSTI-based regimens [59]. These observations suggest that viral transcription, protein production, and even defective viral particles produced by defective proviruses [27–30], might trigger immune responses or viral cytopathic effects, contributing to the rapid decline of the defective proviral DNA during early ART. Interestingly, in the absence of therapy, we have shown that defective proviral DNA copy numbers, rather than the size of the intact viral reservoir, is significantly associated with viral load, cell-associated msRNA, and CD4^+^ count cell [31].

Following this initial decline, we observed a stabilization of the defective proviral DNA over the subsequent 20 years of ART. Similar patterns of early stabilization in the defective proviral reservoir have been reported by Barbehenn *et al.*, who observe stabilization after 5 weeks of ART in CD4^+^ T cells [34], and Buchholtz *et al.*, who describe stabilization after 48 weeks of ART in PBMCs [32]. Peluso *et al.* report a slow decline in the defective proviral DNA in CD4^+^ T cells with a half-life of 17.1 years from 617 days to 7 years after ART initiation, which stabilizes thereafter [35]. Additionally, Gandhi *et al.* describe a stable copy number of defective proviral DNA in CD4^+^ T cells between 7.1 and 12 years after ART initiation [39]. Together, these findings indicate that after the initial phase, the number of defective viral DNA copies stabilizes, suggesting a balance between cell proliferation and cell death, whereas the intact viral reservoir, although at a lower rate, continuous to decline.

After the first decade of ART, we observed that also the decline of the intact reservoir eventually stabilizes in PBMCs. Similarly, within the previous discussed study of McMyn *et al.*[36] and a follow-up study of Gandhi *et al.*[40], it was found that the intact reservoir size levels off or can even increase after 5–10 years of ART. Additionally, the study of Peluso *et al.* reports substantial variability in the decline of the intact reservoir between individuals, of with some even showing expansions [35]. In line with other studies, we found no evidence of viral persistence via ongoing viral replication, as evidenced by the lack of viral evolution [3,5,7,8]. Instead, this suggests that the loss of infected cells is compensated by cell proliferation [60,61]. Clonal proliferation of infected cells on ART has been documented in several studies [62–64], and a recent study showed that, even after two decades of ART, the proviral reservoir predominantly consisted of HIV-infected cellular clones, resulting in reduced HIV diversity [65]. Following this, we observed larger proviral reservoirs in memory T cell subsets compared to naive T cells 20 years after treatment initiation, especially within the EM and CM cells, likely due to the higher proliferation potential of memory T cells [17–20]. Other studies similarly report an enrichment of the HIV reservoir in memory T cells [12,14,15,22], with clonality increasing as cells become more differentiated [14]. Together, these studies suggest that the persistence of the viral reservoir decades after ART is a maintained by cellular proliferation, which can even lead to an increase in the intact viral reservoir. This cellular proliferation is known to be driven by homeostatic mechanisms or clonal expansions triggered by antigens or integration sites [15,62,66,67]. Interestingly, we did not observe the presence of massive clonal proliferation, as indicated by the lack of identical *env*-clones within memory subsets decades after ART initiation in these individuals. Therefore, we suggest that besides clonal proliferation, homeostatic proliferation and cellular differentiation, as supported by other studies [14,15], also plays an important role in the persistence of the proviral reservoir decades after ART.

Although other studies have demonstrated that CXCR4-using viral strains are more prevalent in naive T cells as compared to memory cells [52,56], we were unable to distinguish both populations based on coreceptor usage and therefore could not further assess the role of T cell differentiation as a mechanism of cellular proliferation in HIV persistence. The changes in FPR levels due to cellular differentiation on ART might also be too subtle to detect. Nevertheless, Gartner *et al.* observed higher percentages of identical sequences in the most differentiated effector memory subsets as compared to the other subsets during the first decade of ART [56], highlighting the role of cellular differentiation as a mechanism of HIV persistence decades after ART. Additionally, while naive cells showed the lowest levels of absolute numbers of the intact and defective reservoirs, the relative fraction of intact viruses was larger as compared to memory cells. This has been observed before in people with chronic HIV infections [68]. The relatively long half-life of the naive cells might therefore also significantly contribute to the persistence of the intact reservoir over time. Future studies examining detailed next-generation sequencing across multiple time points after ART are needed to further investigate clonal expansion dynamics within *env* sequences over time and among T cell subsets.

We are aware of the limitations of this study which predominantly includes its relatively small study size, and limited cell numbers especially at baseline and during the early timepoints on ART. Consequently, this limited the detection of proviral DNA copies and the depth of HIV *env* sequencing to further study clonality related to the integration-sites of infected cells in more detail. Moreover, the lack of complete paired samples across individuals, combined with the small sample size, limited our ability to conducts paired analyses on the full cohort or perform linear mixed effect modelling to study subtle changes in dynamics over time. Additionally, due to limited cell availability, we could not conduct quantitative viral outgrowth assays (QVOA) and relied on IPDA measurements as an indication of the replication-competent reservoir. Another limitation of our study was the exclusive inclusion of men in our cohort, which prevents us from addressing potential differences in HIV reservoir dynamics between men and women. This is particularly important as previous studies have shown that HIV reservoir dynamics can differ between sexes [69,70].

## Conclusion

Altogether, our data contribute to the understanding of the dynamics and persistence of the proviral reservoir during two decades of ART. We show that peripheral cells containing the intact proviral DNA are selectively lost during the first decade of suppressive ART, followed by a decade of stabilization of this reservoir in the memory T cell subsets. In the absence of clear signs of viral evolution and massive clonal expansion, homeostatic proliferation might be a key driver of HIV persistence during long-term ART.

## Acknowledgements

The authors thank all the participating individuals for their selfless contribution to this study.

Funding: The original trial is supported by Roche, participant materials derived from this trial are used for this study. The study is supported by funding from the Aidsfonds (P-2013034) and Health-Holland (LSHM19100-SGF & LSHM19101-SGF).

Author contributions: A.W., M.N., J.A. and A.H. designed the study. M.M.N., K.B., D.J., T.K., N.B. and S.O. performed experiments. M.M.N., T.H., W.S., L.G. and E.L.E. performed analyses under the supervision of K.T., A.P., R.B., J.S., J.B. and M.N. M.M.N. and K.B. wrote the manuscript, and all authors approved it.

Data availability: Envelope sequences are available upon request.

### Conflicts of interest

The authors do not declare any competing interests related to this study.

## Supplementary Material

Supplemental Digital Content

## Supplementary Material

Supplemental Digital Content
