# Supplementary material for: Selective decline of intact HIV reservoirs during the first decade of ART followed by stabilization in memory T cell subsets
Source: AIDS. 2025 Feb 20;39(7):798–811. doi: 10.1097/QAD.0000000000004160 (PMC12077340; doi:10.1097/QAD.0000000000004160)

**Supplementary Figure 1. Dynamics of HIV Psi and ENV proviral DNA during two decades of ART. A)**  *Size of the* ***A)*** *psi and* ***B)*** *env proviral DNA as measured via the IPDA. Data points below the lower bound of detection (open squares) or quantified with less than 95% reliability (triangles) are indicated. Horizontal x-axis is not in scale; “w” represents time points weeks, “y” represents years after ART. Statistical comparisons of the Wilcoxon singed-rank tests are shown. With P-values <0.1, these comparisons were additionally tested with a Mann-Whitney U test (unpaired). P-values of the Wilcoxon singed-rank test <0.1 and the Mann-Whitney U test <0.05 are indicated in bold. The sum of the proviral psi and env copies are considered as the total number of defective proviral DNA copies and is visualized in* ***Figure 1C****.*


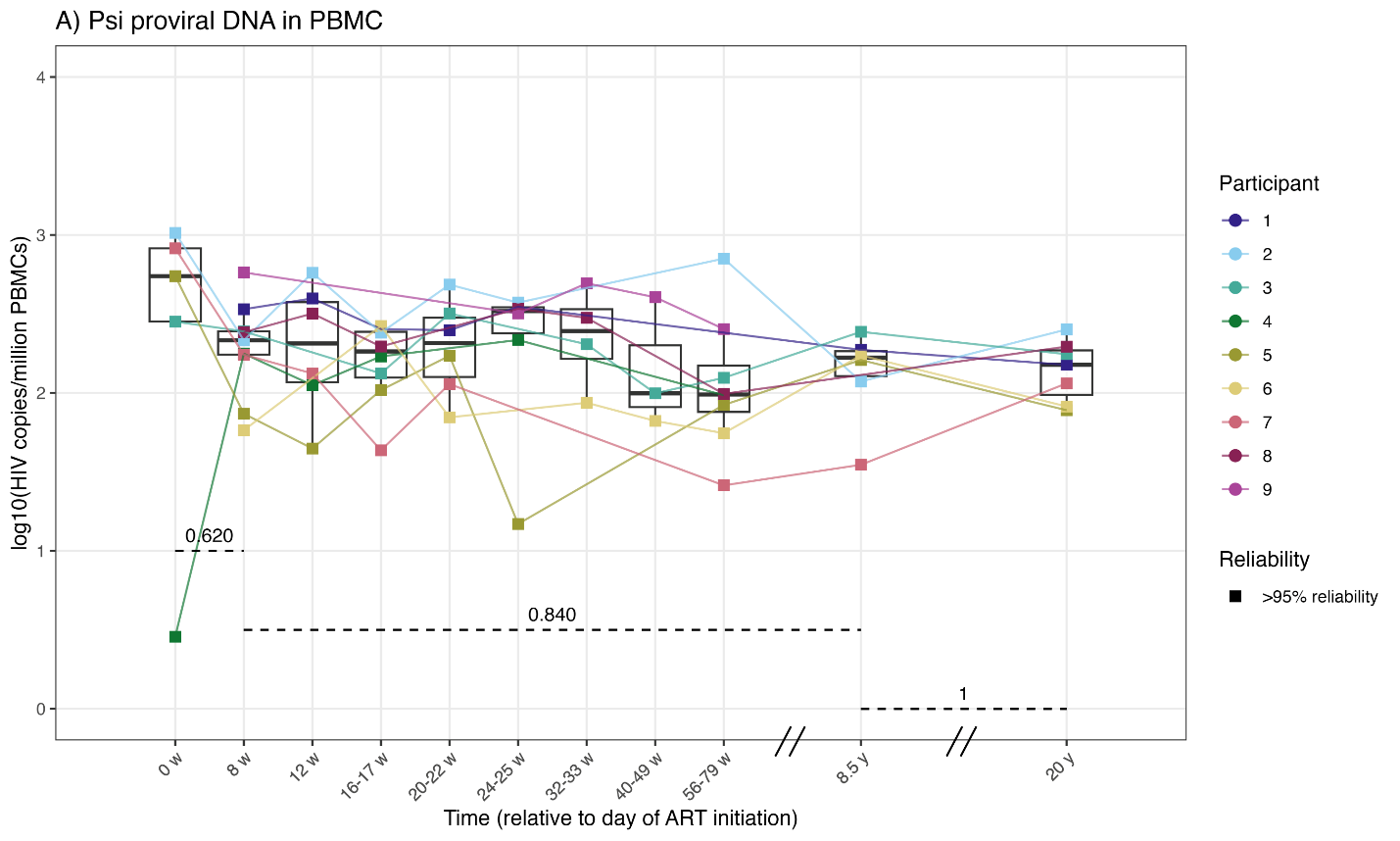


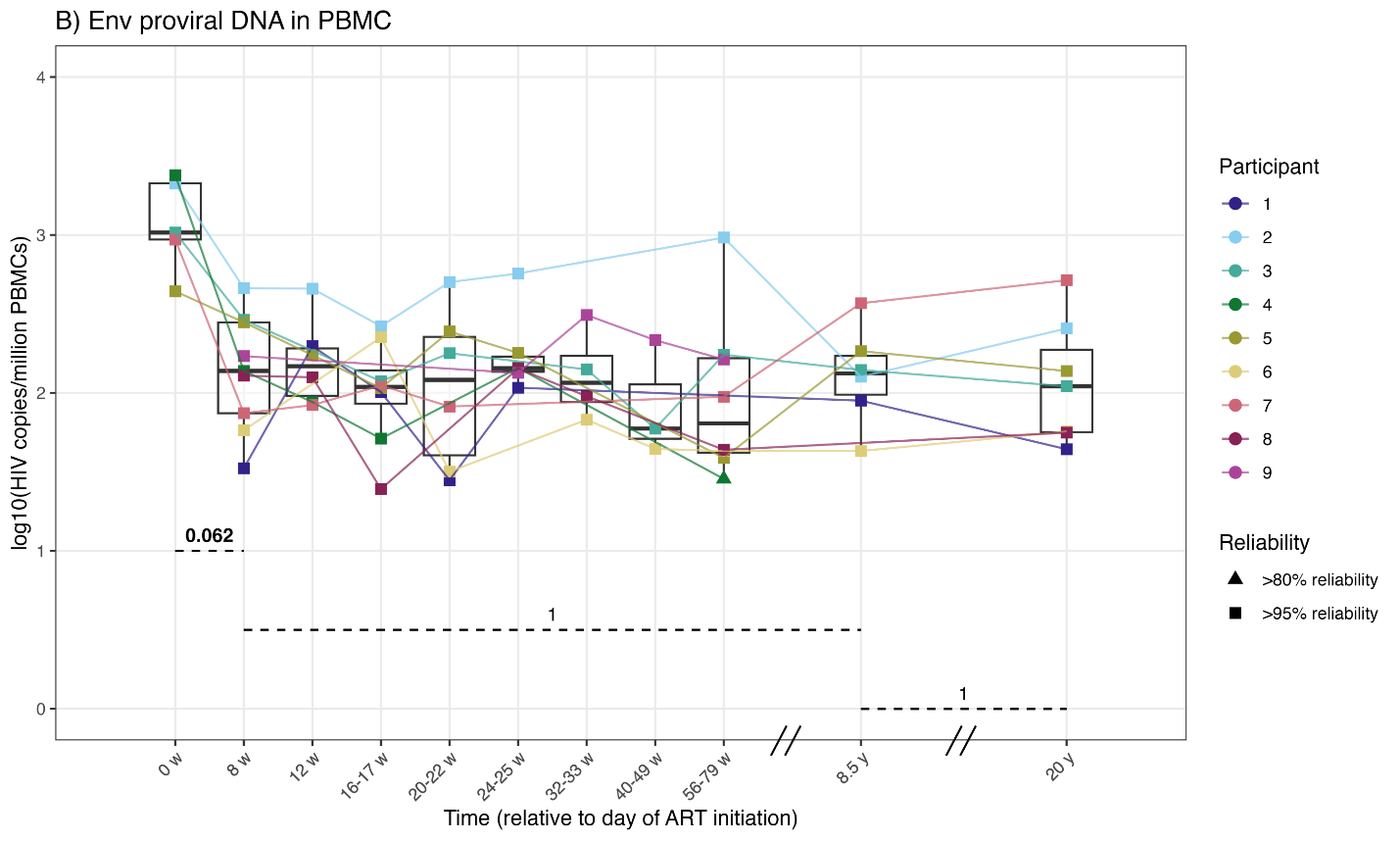


**Supplementary Figure 2. Relative fraction of the intact proviral reservoir.** *The size of the intact and defective proviral DNA was measured via the IPDA. The relative intact proviral fraction represents the percentage of the intact reservoir relative to the total proviral DNA as measured via the IPDA. Data points below the upper bound of detection (open square) or quantified with less than 95% reliability (triangle) were indicated. Statistical comparisons over time periods were performed via Wilcoxon singed-rank test. With P-values* *<0.1, these comparisons were additionally tested with the Mann-Whitney U test (unpaired). P-values of the Wilcoxon singed-rank test <0.1 and the Mann-Whitney U test <0.05 are indicated in bold.*


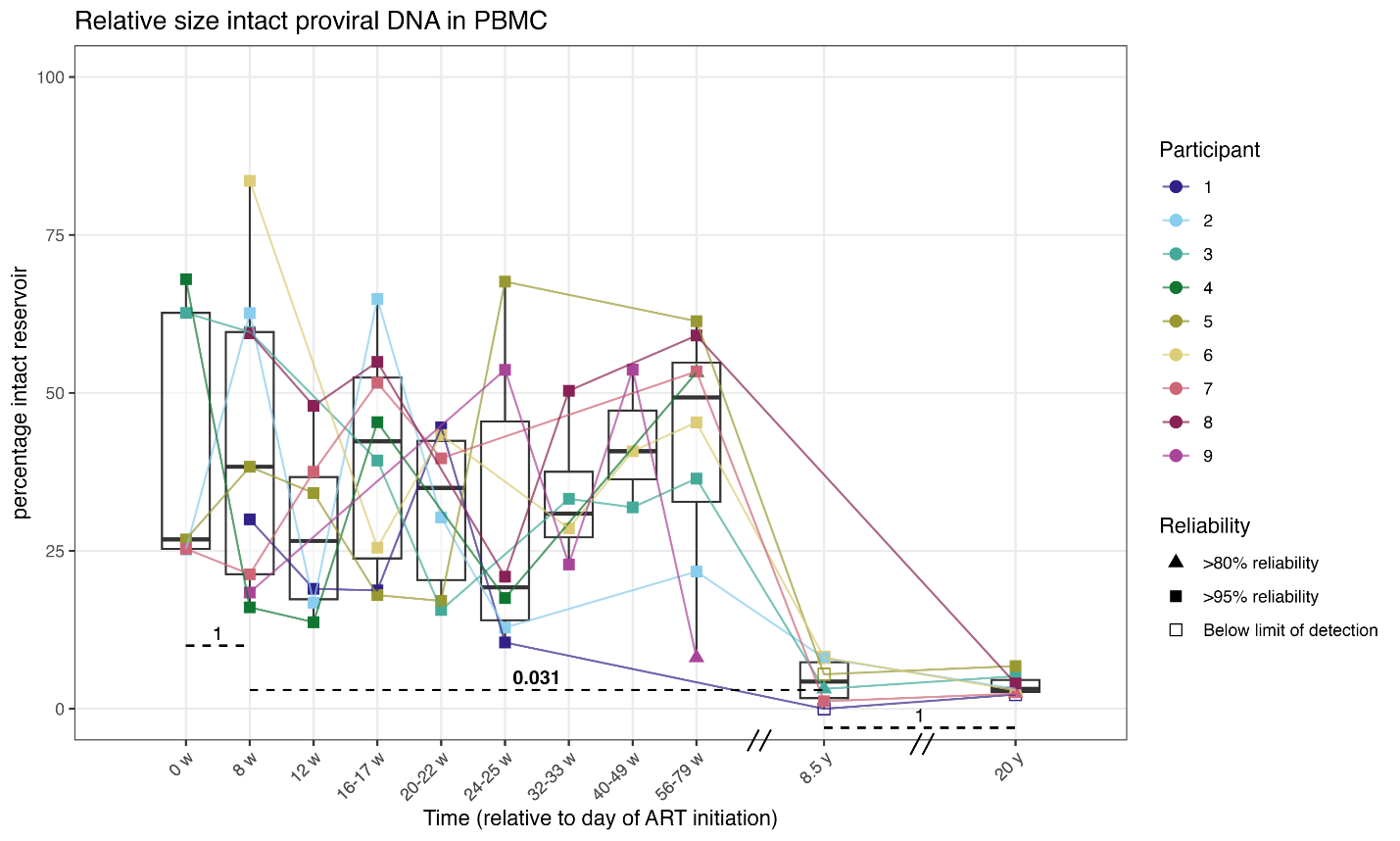


***Supplementary Figures 3A-H. Violin plots of the FPR values for each individual in the PBMCs and different CD4^+^ T cell-subsets at 8.5- and 20-years post treatment initiation.***  *The weighted occurrence of each unique V3 amino acid sequence was considered when evaluating the FPR.*


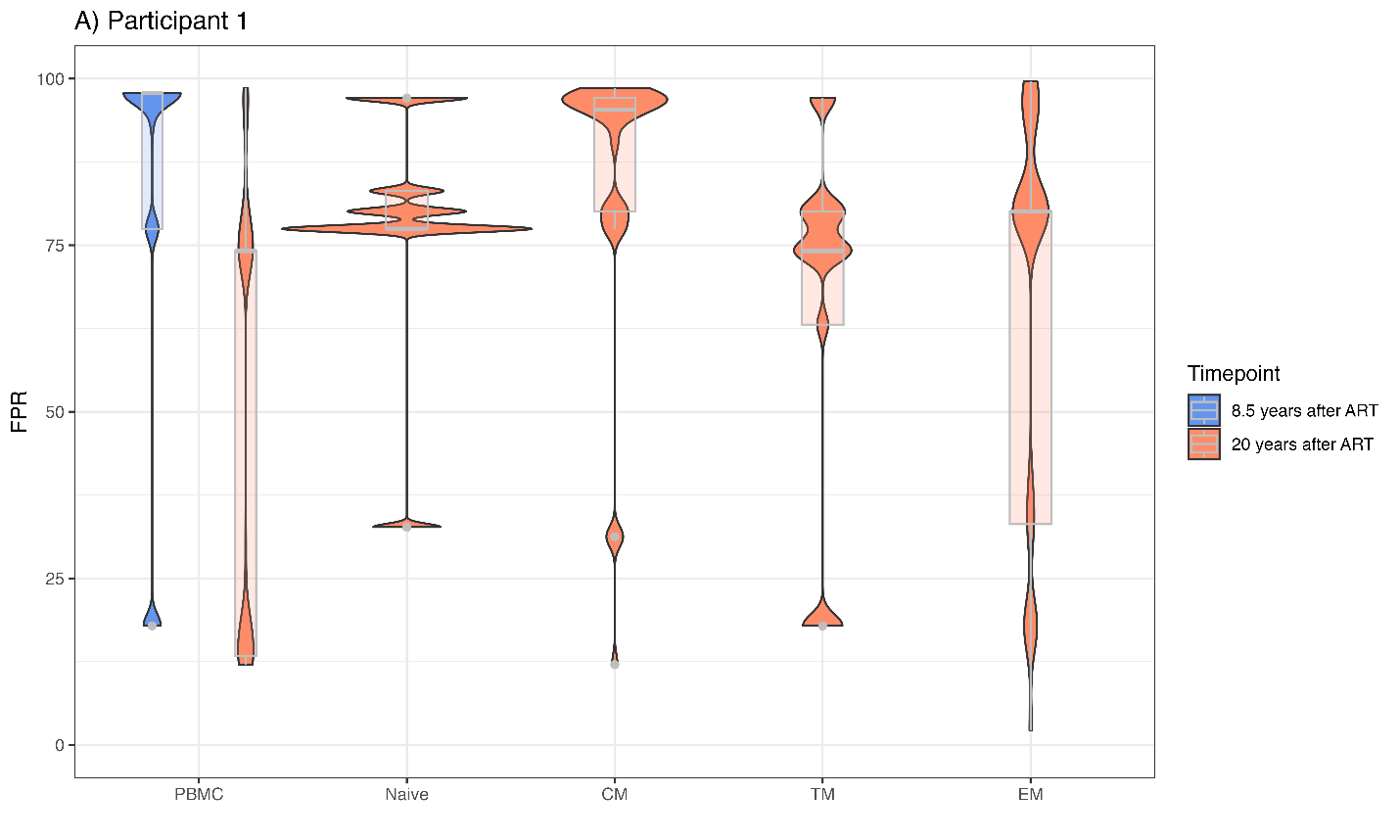


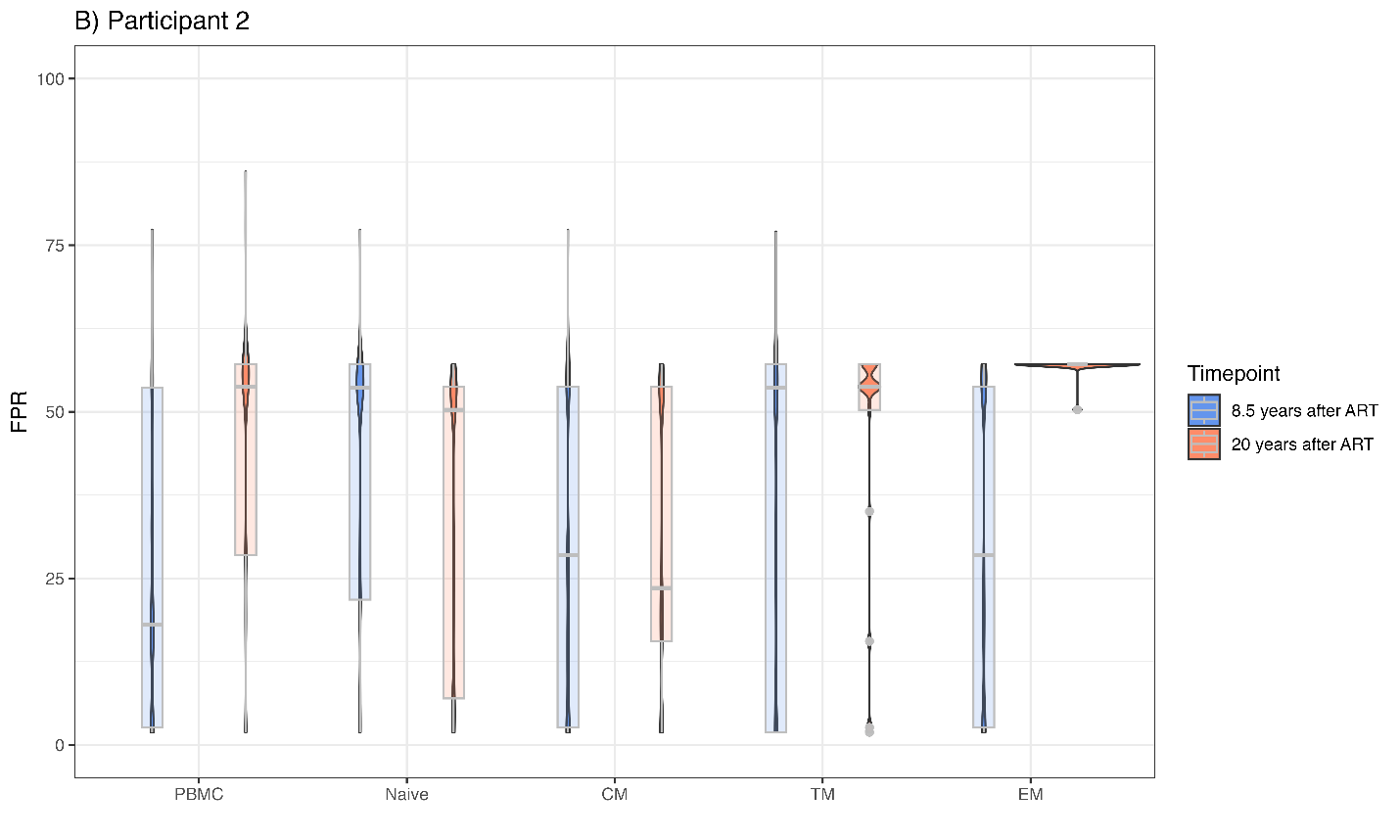


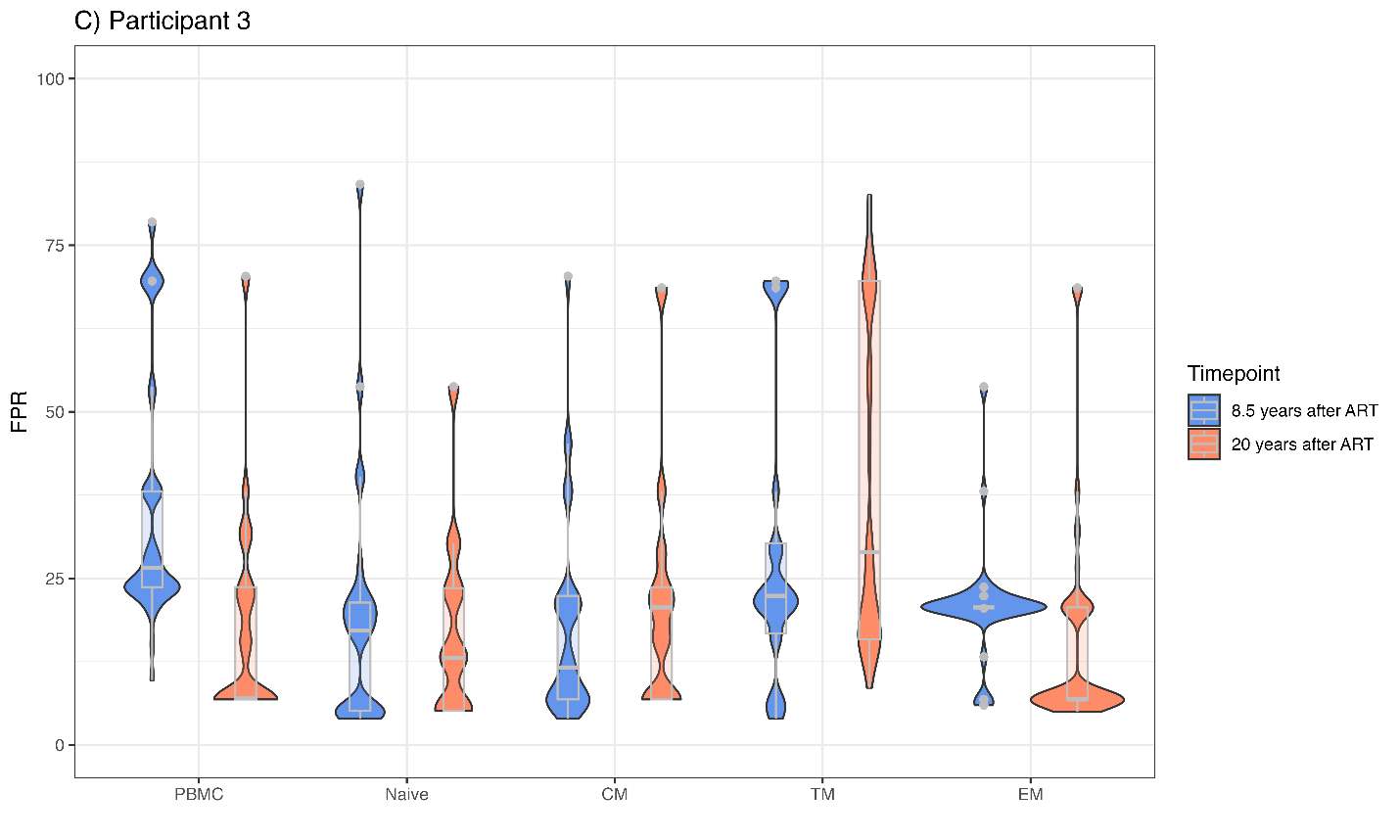


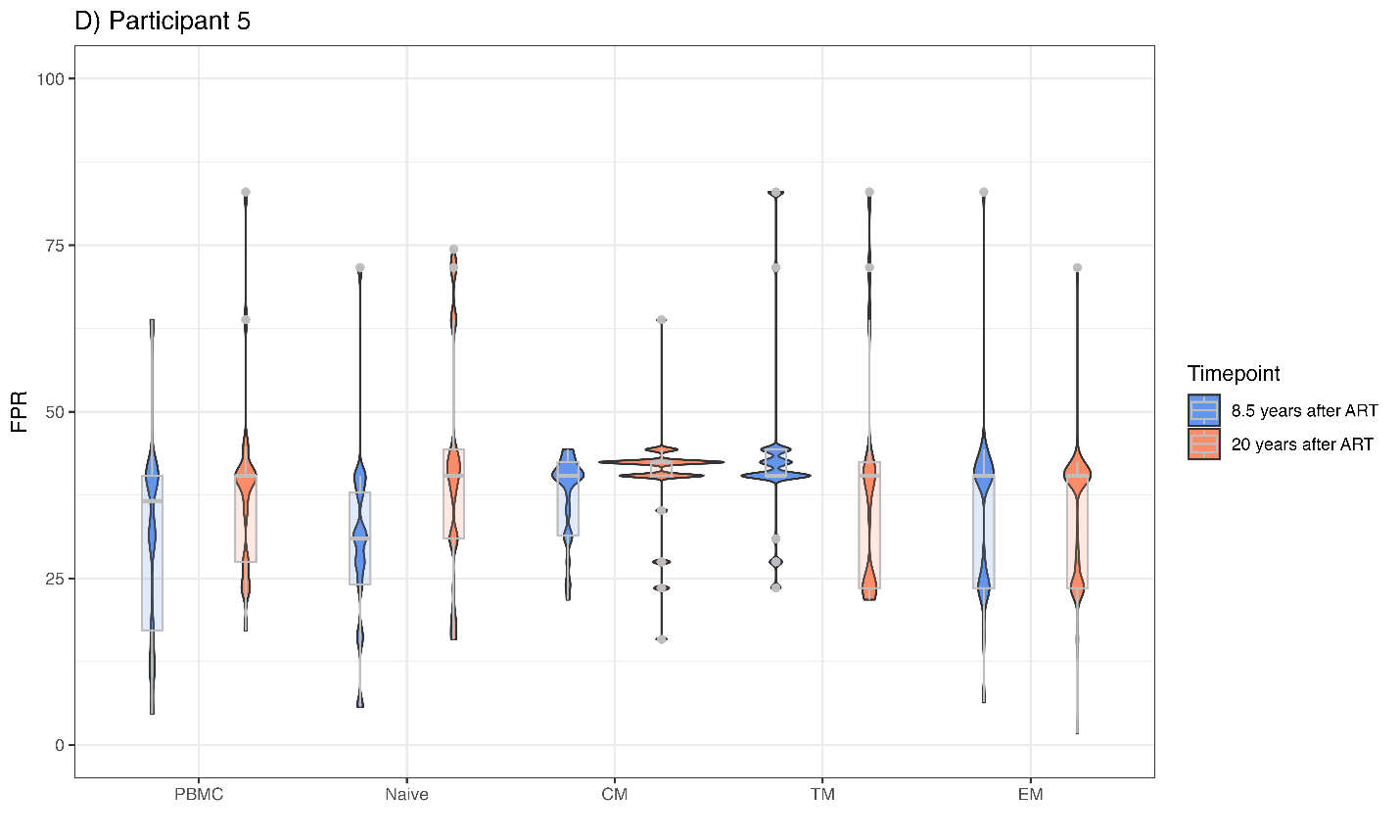


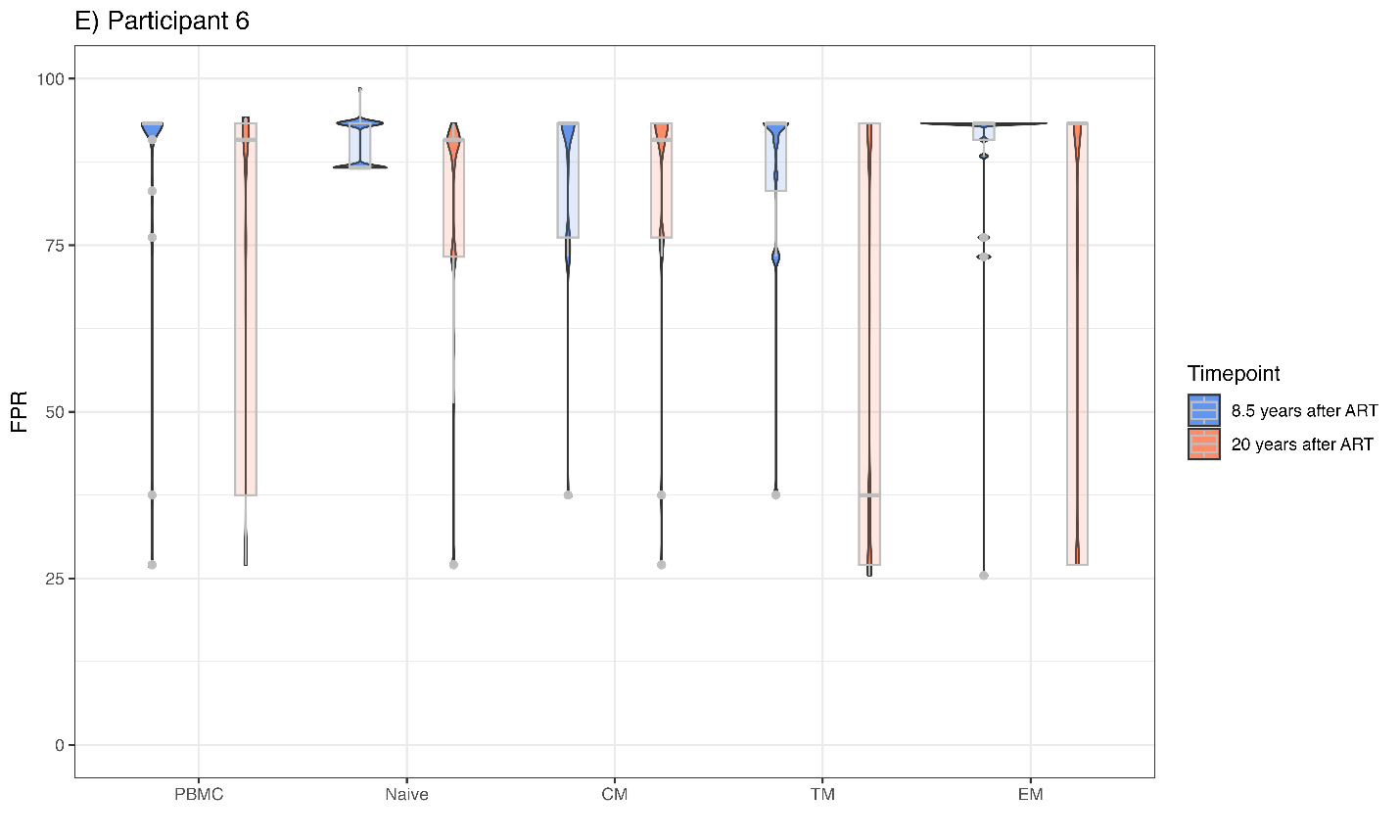


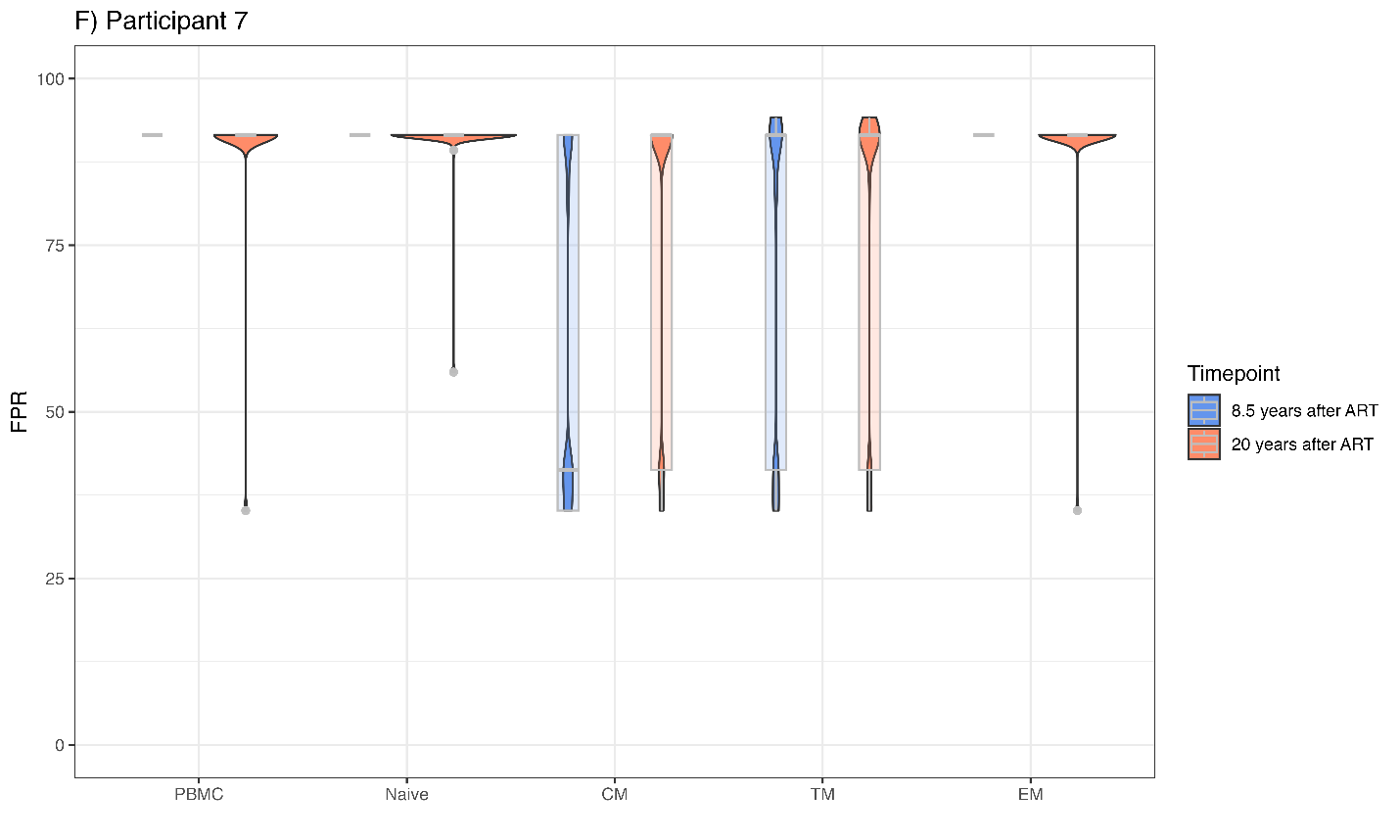


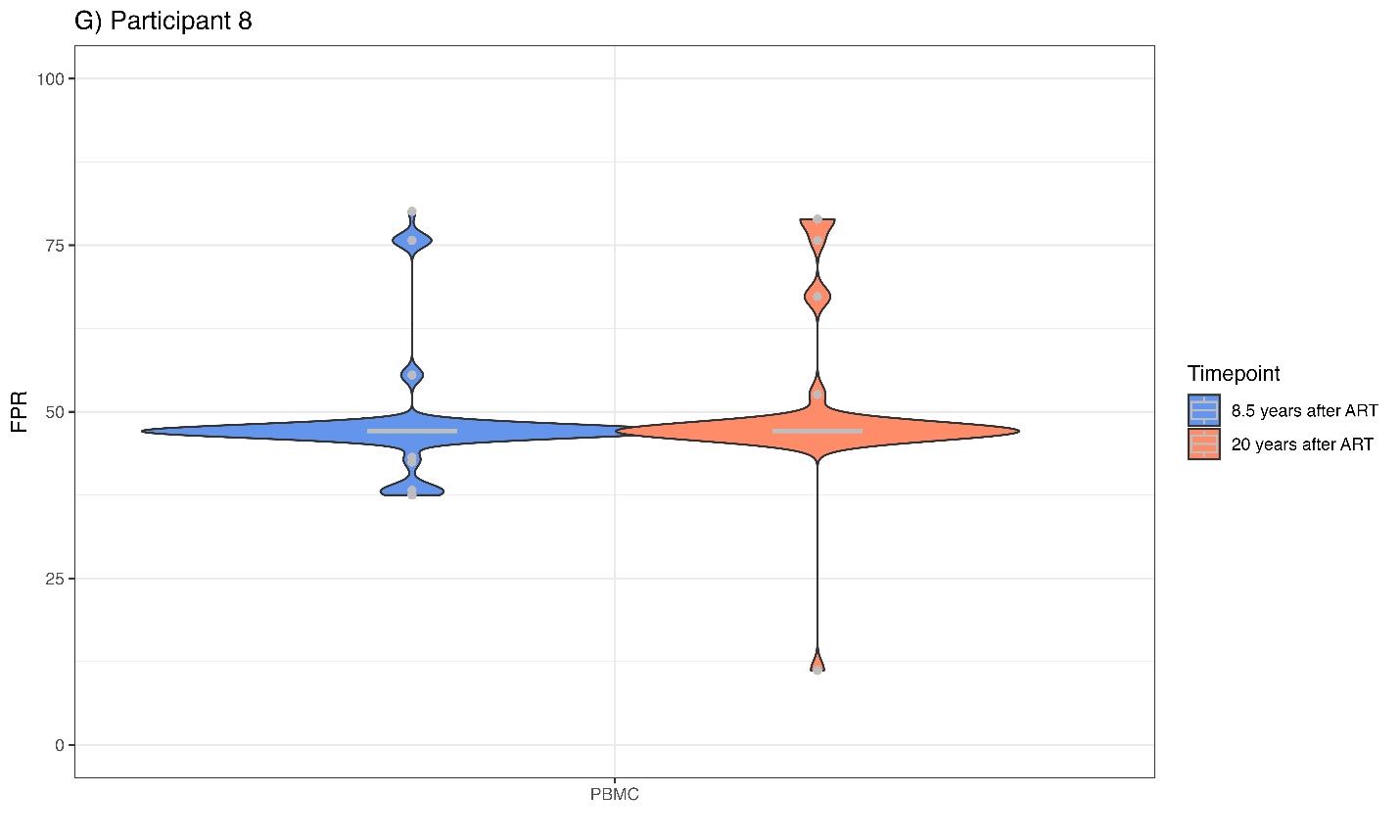


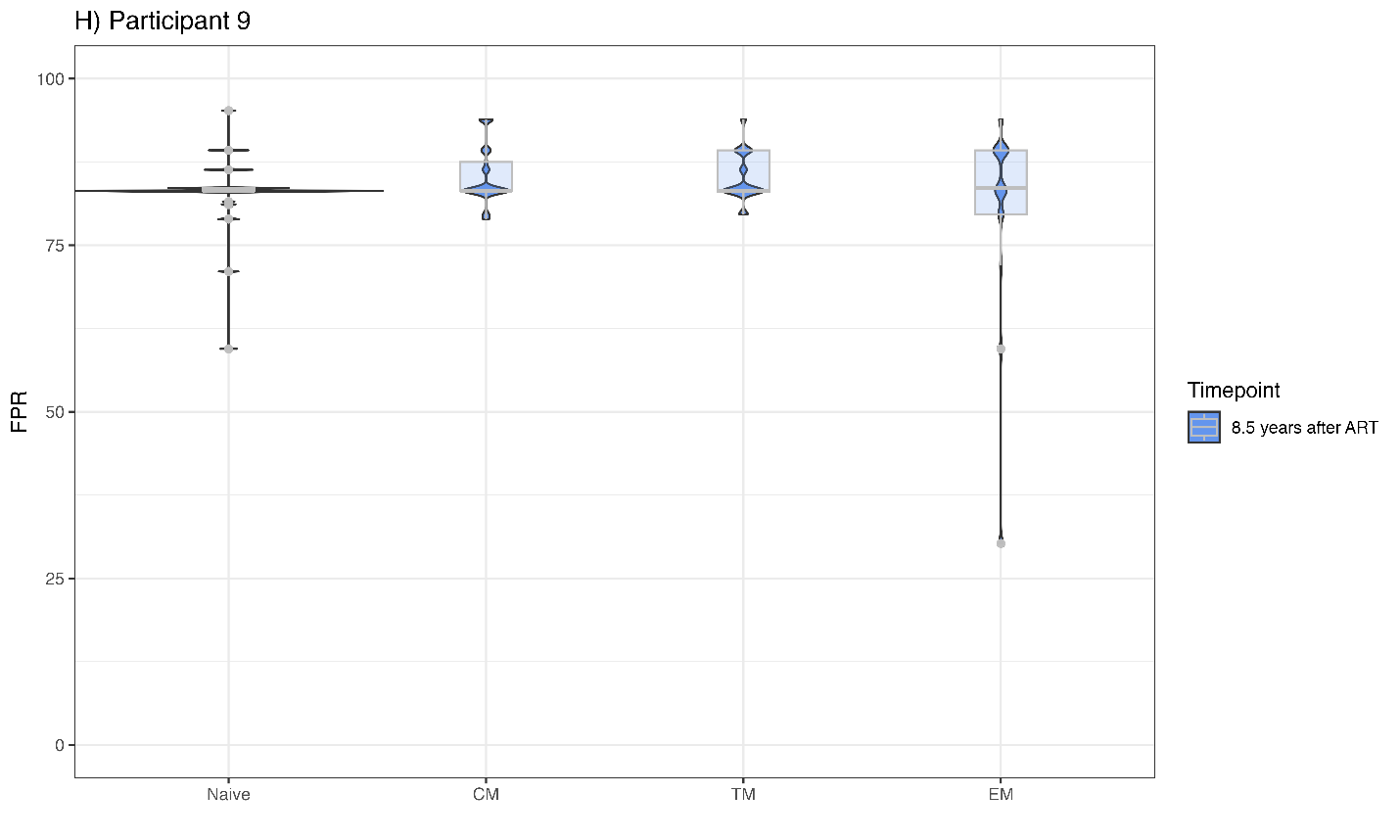


**Supplementary Figure 4. Phylogenetic trees of unique V3 sequences over two decades of ART.** *Number of sequences were* ***A)*** *n=113 for individual 1,* ***B)*** *n=200 for individual 2,* ***C)*** *n= 193 for individual 3,* ***D)*** *n=48 for individual 4,* ***E)*** *n= 196 for individual 5,* ***F)*** *n=134 for individual 6,* ***G)*** *n= 87 for individual 7,* ***H)*** *n= 19 for individual 8, and* ***I)*** *n= 52 for individual 9. Replicate samples were pooled. Pre-treatment RNA sequences were shown in red, sequences from between pre-ART up to 2 years post-initiation were shown in purple, sequences from the 8.5-years’ time point were shown in blue and sequences from the 20-year time point were shown in green. Bootstrap support was indicated in percentage of total bootstraps (100). Sample nomenclature is abbreviated using the format; ‘ years (y) or weeks after (w) or before ART (wn)’, ‘plasma RNA (r), PBMCs (p), naive (n), central memory (c), transitional memory (t), or effector memory (e)’, and ‘replicate number’.*


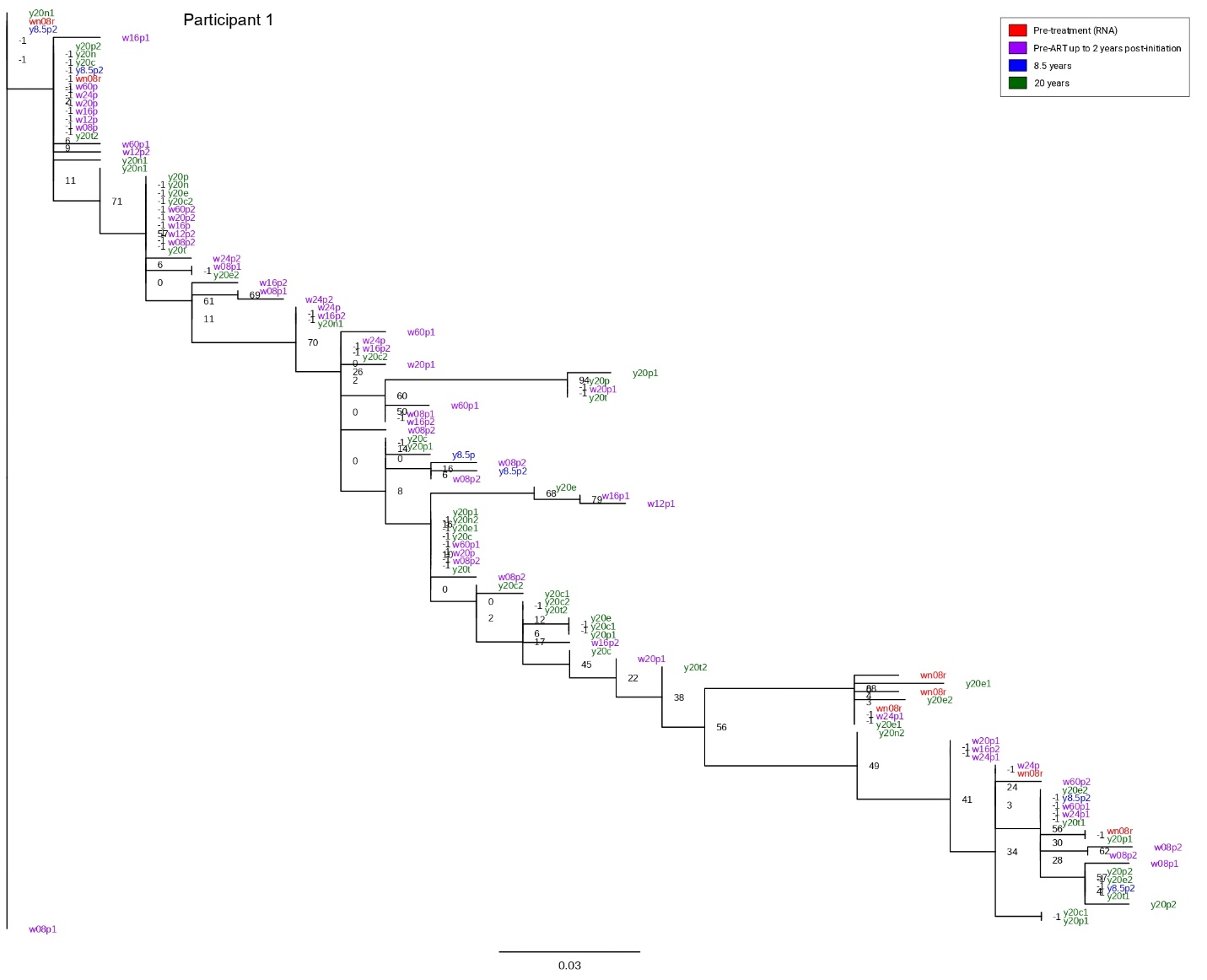


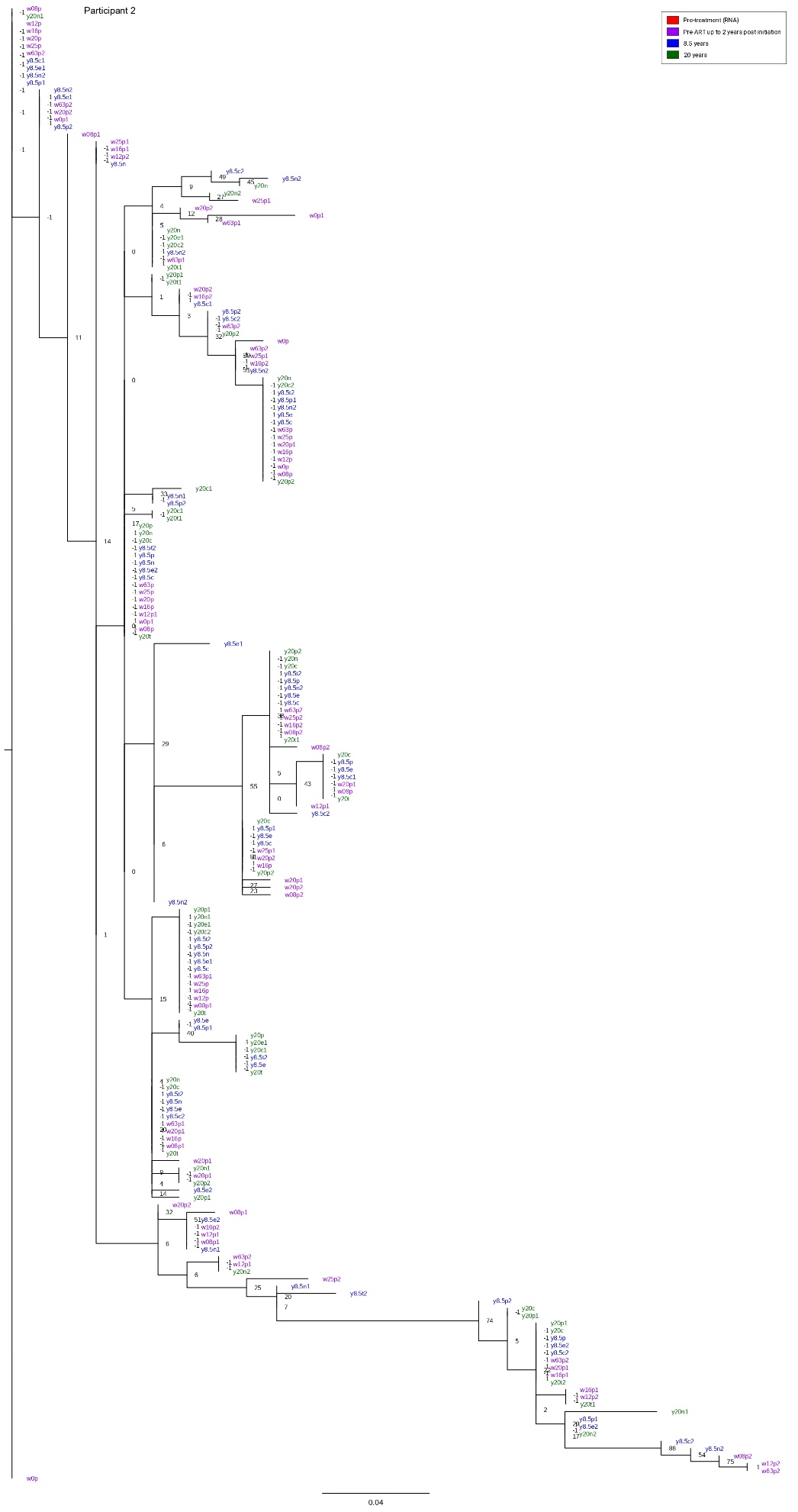


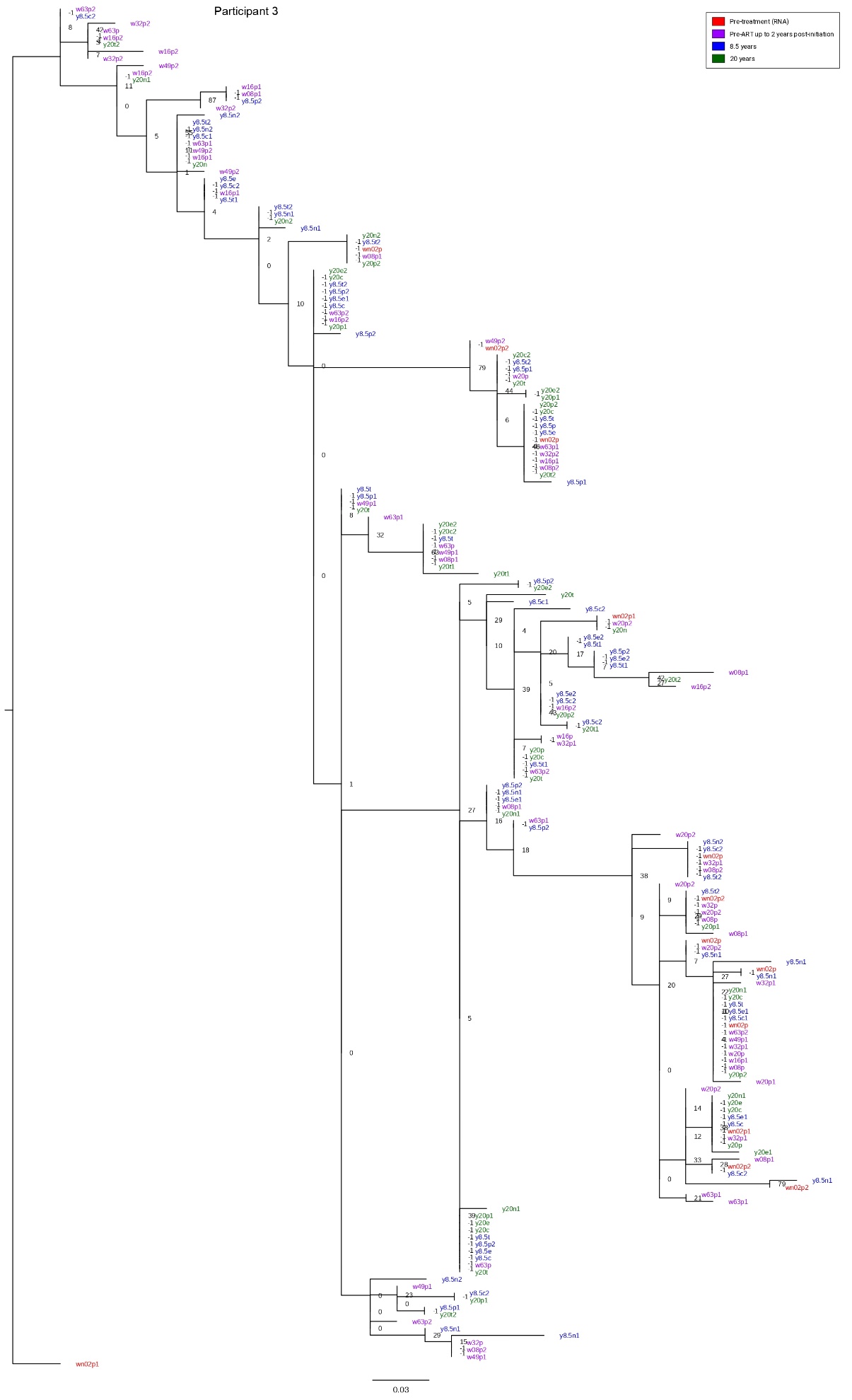


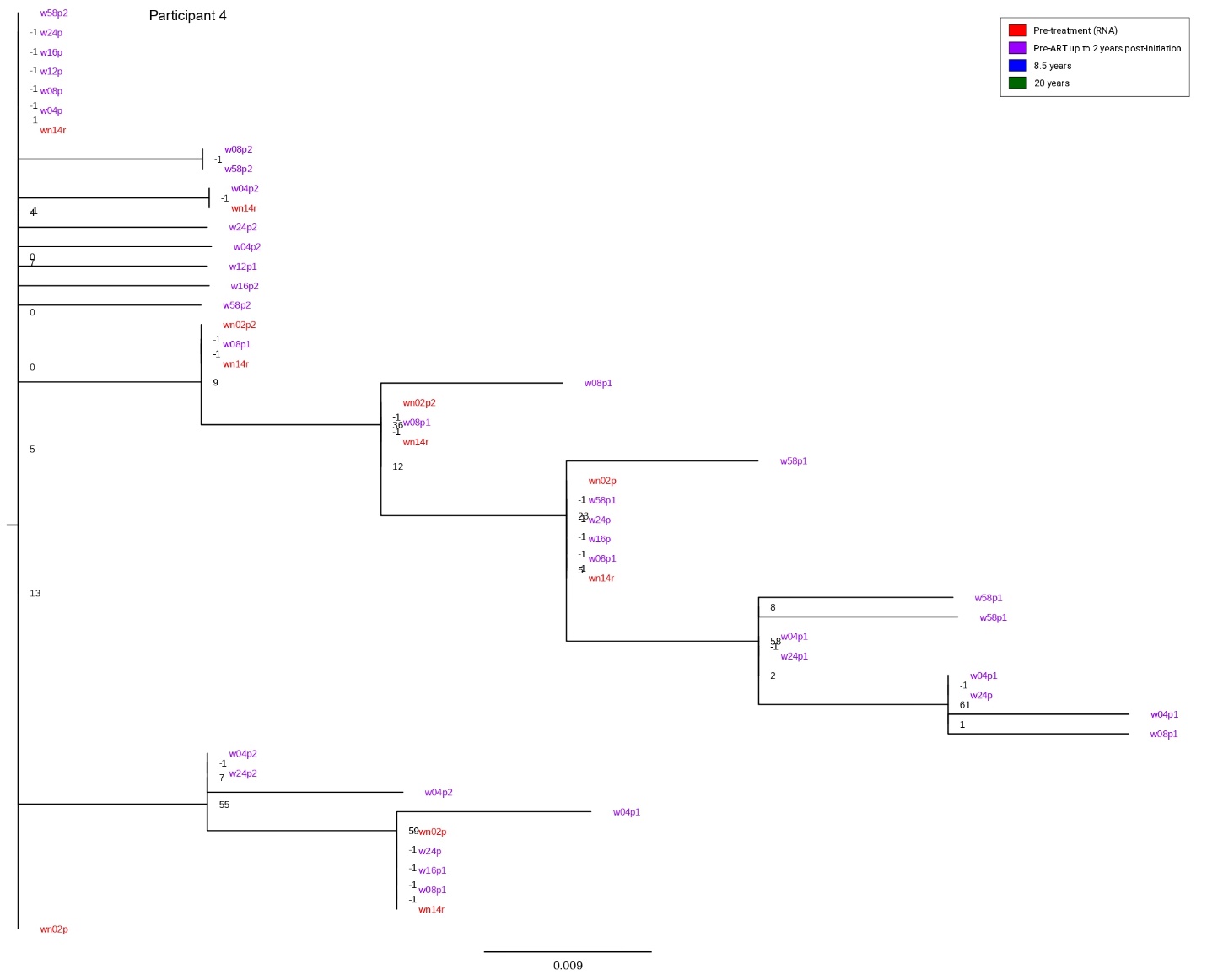


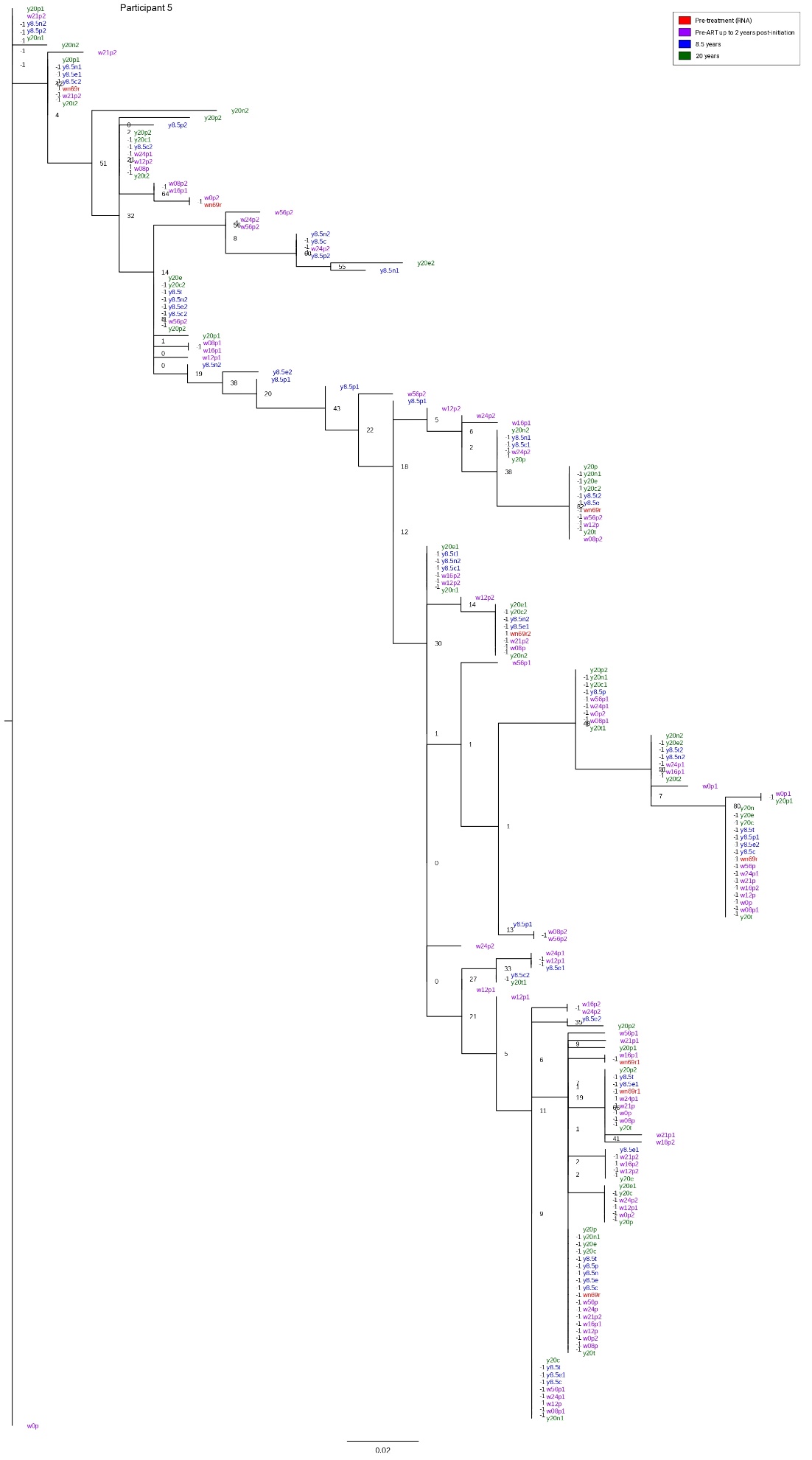


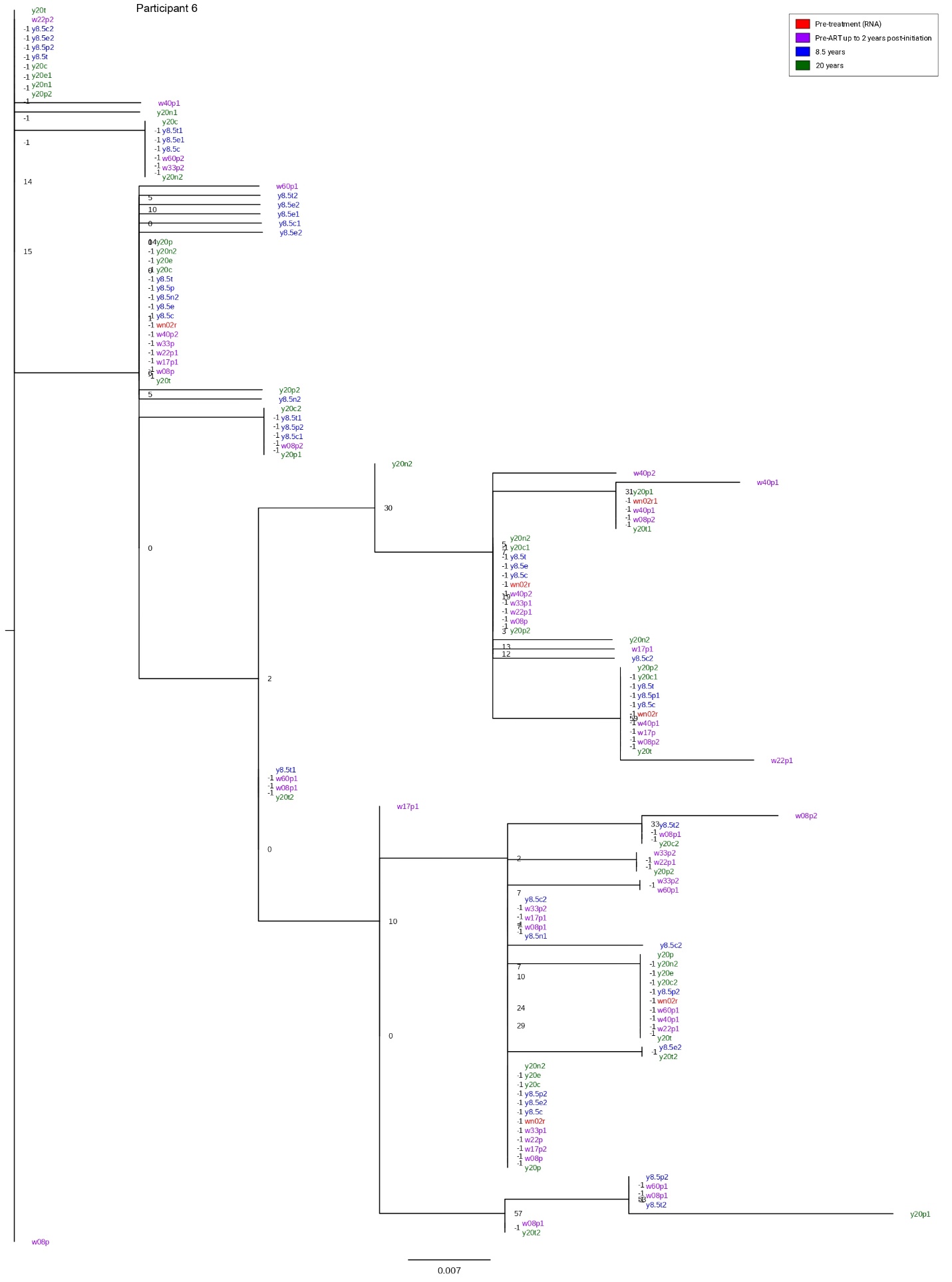


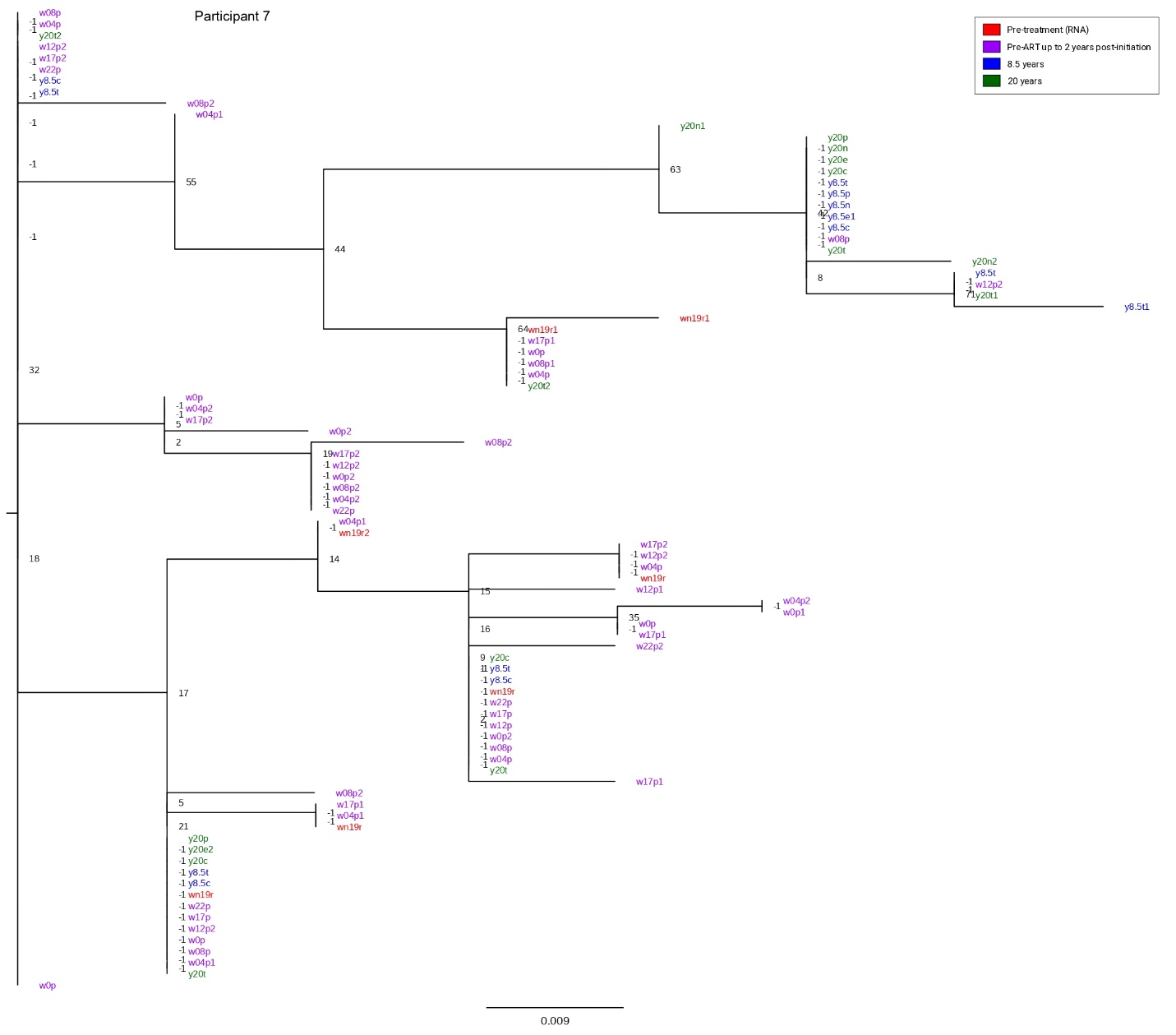


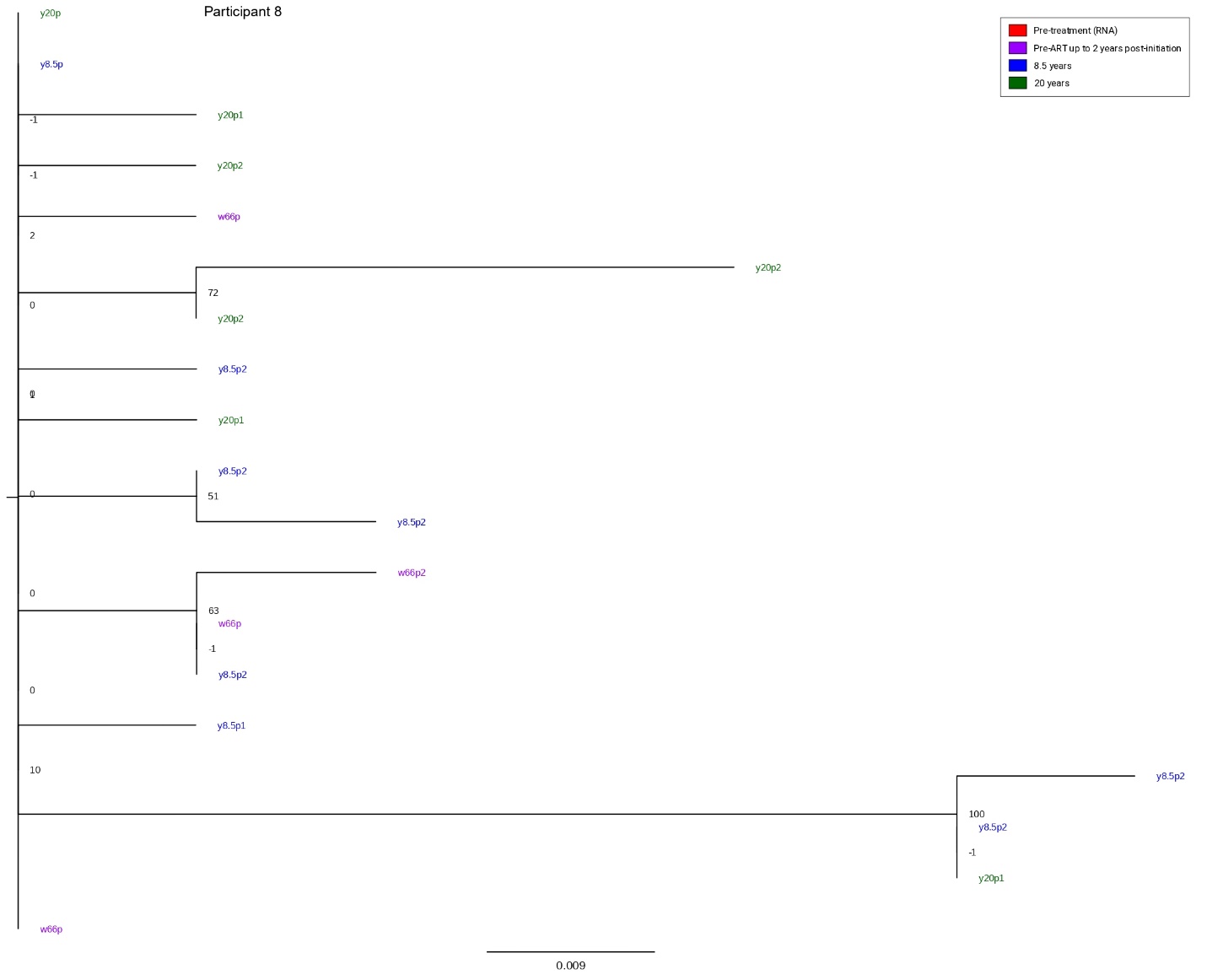


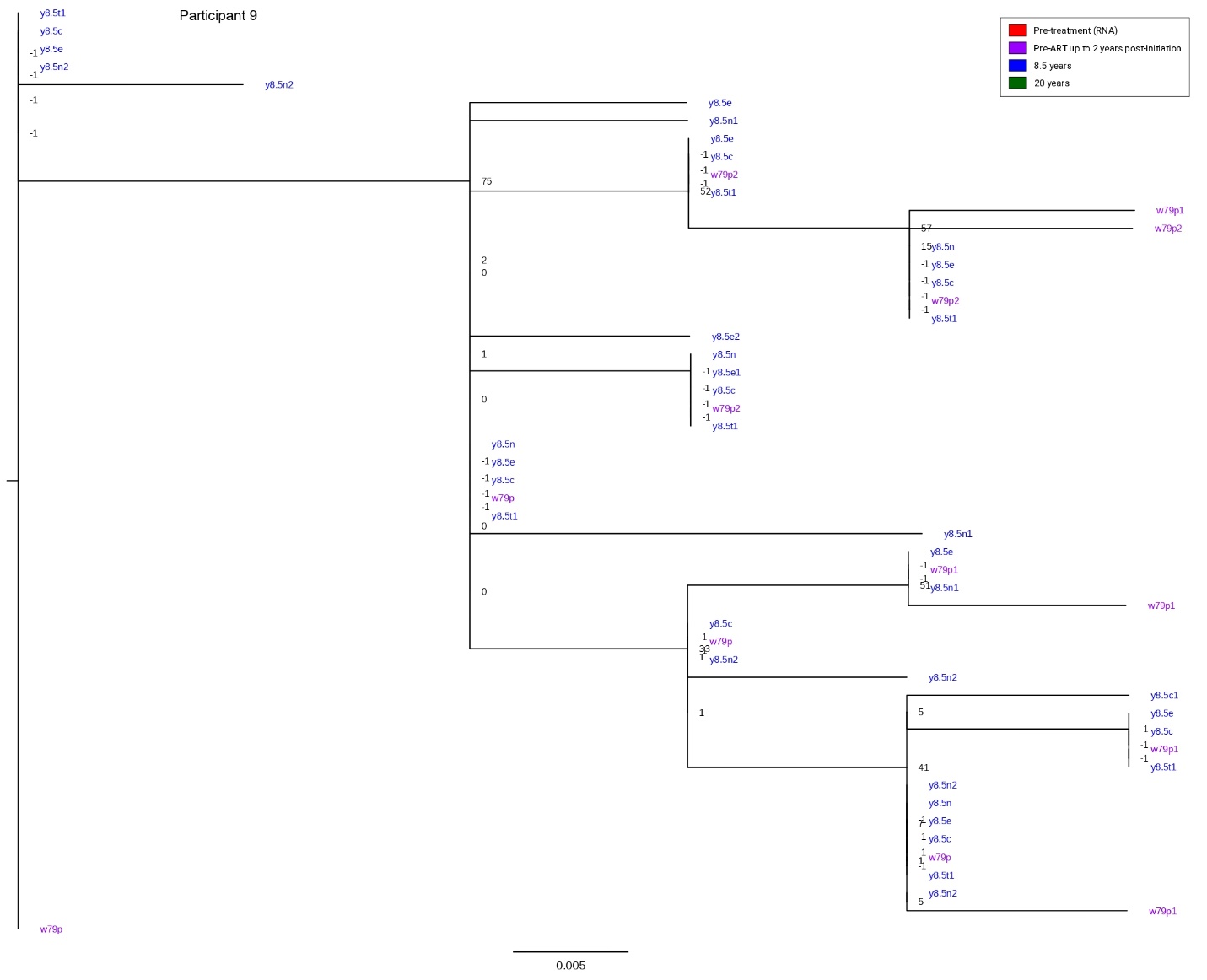

Supplement: Supplemental Digital Content [file aids-39-798-s001.docx]
