# Supplementary material for: Selective decline of intact HIV reservoirs during the first decade of ART followed by stabilization in memory T cell subsets
Source: AIDS. 2025 Feb 20;39(7):798–811. doi: 10.1097/QAD.0000000000004160 (PMC12077340; doi:10.1097/QAD.0000000000004160)
Supplement: Supplemental Digital Content [file aids-39-798-s002.docx]

## Supplementary Tables

**Supplementary Table 1. Thermal cycle protocol of the ddPCR**

| **Cycling step** | **Temperature (°C)** | **Time** | **#cycles** | **Ramp rate** |
| --- | --- | --- | --- | --- |
| Enzyme activation | 95 | 10 minutes | 1 | ~ 2°c/seconds |
| Denaturation | 94 | 30 seconds | 40 |  |
| Annealing/extension | 60 | 1 minute |  |  |
| Enzyme deactivation | 98 | 10 minutes | 1 |  |

**Supplementary Table 2. Cycling conditions ddPCR LTR**

| **Primer or probe** | **Function** | **Sequence** | **Concentration** | **Target HXB2** |
| --- | --- | --- | --- | --- |
| HIV-LTR S4 | Forward primer | AAG CCT CAA TAA AGC TTG CCT TGA | 900nmol/L | 520–543 |
| 3'UNI-KS-6 | Reverse primer 1 | GAG GGA TCT CTA GTT ACC AGA GTC ACA | 900nmol/L | 574–600 |
| 3'UNI-KS-6-AG | Reverse primer 2 | GAG GGA TCT CTA GTT ACC AGA GTC CTA | 900nmol/L | 574–600 |
| LTR FAM- probe | MGB probe | TAG TGT GTG CCC GTC TG | 300nmol/L | 554–570 |
| RPP30-F | Forward primer | AGA TTT GGA CCT GCG AGC G | 400nmol/L | n/a |
| RPP30-R | Reverse primer | GAG CGG CTG TCT CCA CAA GT | 400nmol/L | n/a |
| RPP30 VIC-probe | MGB probe | TTC TGA CCT GAA GGC TCT GCG CG | 300nmol/L | n/a |

**Supplementary Table 3. Inner and outer PCR primers**

| **Primer name** | **Sequence** | **Concentration** | **Target HXB2** |
| --- | --- | --- | --- |
| Outer PCR | | | |
| envF1.1 | GGA TAT AAT CAG YYT ATG GGA | 320 nmol/L | 6542-6562 |
| envR1.1 | GGT GGG TGC TAY TCC YA ITG | 320 nmol/L | 7702-7721 |
| envF1.2 | GAG GAT ATA ATC AGT TTA TGG | 320 nmol/L | 6540-6560 |
| envR1.2 | GGT GGG TGC TAT TCC TAA TGG | 320 nmol/L | 7701-7721 |
| Inner PCR | | | |
| envF2.1 | GAT CAA AGC CTA AAR CCA TGT | 300 nmol/L | 6561-6581 |
| envR2.1 | CTC CAA TTG TCC YTC ATH TYT CC | 300 nmol/L | 7641-7663 |
| envF2.2 | GAT CAA AGC CTA AAG CCA TG | 300 nmol/L | 6561-6580 |
| envR2.2 | ACT TCT CCA ATT GTC CCT CAT AT | 300 nmol/L | 7645-7667 |

**Supplementary Table 4**. **Inner and outer PCR cycling conditions**

| **Cycling step** | **Temperature** | **Time** | **#cycles** |
| --- | --- | --- | --- |
| **Outer PCR Cycling conditions** | | | |
| Reverse transcription* | 45°C | 45 minutes | 1 |
| Enzyme activation | 94°C | 2 minutes | 1 |
| Denaturation 1 | 94°C | 30 seconds | 10 |
| Annealing 1 | 45°C | 30 seconds |  |
| Extension 1 | 68°C | 3 minutes |  |
| Denaturation 2 | 94°C | 30 seconds | 10 |
| Annealing 2 | 50°C | 30 seconds |  |
| Extension 2 | 68°C | 4 minutes |  |
| Denaturation 3 | 94°C | 30 seconds | 15 |
| Annealing 3 | 55°C | 30 seconds |  |
| Extension 3 | 68°C | 5 minutes |  |
| Extended extension | 68°C | 7 minutes | 1 |
| **Inner PCR Cycling conditions** | | | |
| Enzyme activation | 94°C | 1 minute | 1 |
| Denaturation 1 | 94°C | 30 seconds | 30 |
| Annealing 1 | 50°C | 30 seconds |  |
| Extension 1 | 72°C | 90 seconds |  |
| Extended extension | 72°C | 7 minutes |  |

**The first reverse transcription step was only performed for RNA samples.*

**Supplementary Table 5**. **Overview of ART regimens and major clinical events**

| **ID** | **Gender** | **Start ART (mm-dd-yy)** | **ART initiation** | **ART at 7-10 years after start therapy** | **ART at 18-20 years after start therapy** | **Clinical events during study** |
| --- | --- | --- | --- | --- | --- | --- |
| **1** | M | 01-22-97 | AZT+3TC+SQV | AZT+3TC+NVP | FTC+TDF+NVP | 2009: lipodystrophy, 2013: hypertension |
| **2** | M | 05-28-97 | AZT+3TC+SQV | AZT+3TC+EFV | FTC+TDF+ATV | 2004: neuropathy, 2009: B12 deficiency |
| **3** | M | 05-28-97 | AZT+3TC+IDV | AZT+3TC+EFV | FTC+TDF+EFV | 2009: hypertension, 2010: single dermatome herpes zoster |
| **4** | M | 01-22-97 | AZT+3TC+IDV | Exclusion | Exclusion | 1997: neuropathy |
| **5** | M | 03-04-98 | AZT+3TC+SQV | AZT+3TC+ABC | FTC+TAF+RPV | no major events |
| **6** | M | 01-27-97 | AZT+3TC+SQV | AZT+3TC+LPV/r | FTC+TDF+RPV | 2009: lipodystrophy, 2012: arrhythmia |
| **7** | M | 01-27-97 | AZT+3TC+IDV | AZT+3TC+EFV | FTC+TDF+RPV | 2001: acute appendicitis, 2010: myocardial infarction, 2012: autoimmune hypothyroidism |
| **8** | M | 10-15-97 | AZT+3TC+IDV | AZT+3TC+NVP | FTC+TDF+ATV/r | 1998: neuropathy, 2014: osteoporosis |
| **9** | M | 12-10-97 | AZT+3TC+SQV | AZT+3TC+NVP | Exclusion | 1999: pneumococcal pneumonia |
